# Long-term efficacy and safety of rilpivirine plus abacavir and lamivudine in HIV-1 infected patients with undetectable viral load

**DOI:** 10.1371/journal.pone.0191300

**Published:** 2018-02-16

**Authors:** Nadia Galizzi, Laura Galli, Andrea Poli, Nicola Gianotti, Elisabetta Carini, Alba Bigoloni, Giuseppe Tambussi, Silvia Nozza, Adriano Lazzarin, Antonella Castagna, Daniela Mancusi, Roberta Termini

**Affiliations:** 1 Infectious Diseases, IRCCS San Raffaele, Milan, Italy; 2 Università Vita-Salute San Raffaele, Milan, Italy; 3 Janssen-Cilag SpA, Medical Affairs, Cologno Monzese—Milan, Italy; Universita degli Studi di Roma Tor Vergata, ITALY

## Abstract

**Introduction:**

A regimen with rilpivirine (RPV), abacavir (ABC) and lamivudine (3TC) is simple and may allow the sparing of tenofovir and protease inhibitors. However, data on use of this combination as a strategy of switch are limited. Aims of the study were to assess the long-term efficacy and safety of this regimen.

**Methods:**

Retrospective study on HIV-1 infected patients followed at the Infectious Disease Department of the San Raffaele Scientific Institute, HBsAg-negative, HLA B5701-negative, with no documented resistance to RPV, ABC and 3TC, with HIV-RNA<50 copies/mL who started RPV plus ABC/3TC from March 2013 to September 2015.

The primary outcome was durability [no treatment failure (TF)]. Secondary objectives were to evaluate changes in immunological, metabolic and other safety parameters.

TF was defined as the occurrence of virological failure (VF, 2 consecutive values >50 copies/mL) or discontinuation of any drug in the regimen for any reason.

Patients’ follow-up accrued from the date of RPV plus ABC/3TC initiation to the date of TF (VF or discontinuation of any drug in the regimen) or to the date of last available visit.

Time to TF was evaluated by use of the Kaplan-Meier curves. Mixed linear models were applied to evaluate changes in immunological, metabolic and other safety parameters.

**Results and discussion:**

In this analysis, 100 patients starting RPV plus ABC/3TC were included. By 12, 24 and 36 months after switching to RPV plus ABC/3TC, the proportions of individuals without TF were 88% [95% confidence interval (CI): 79%-93%], 82% (95% CI:73%-89%) and 78% (95% CI:68%-86%), respectively. Time to TF was not significantly influenced by CD4+ nadir (≤200 vs >200 cells/μl; log-rank test: p = 0.311) or pre-ART viral load (<100000 vs ≥100000 copies/mL; log-rank test: p = 0.574) or the type of previous antiretroviral regimen (PI+2NRTIs vs NNRTI+2NRTIs vs Other; log-rank test: p = 0.942).

Over a median follow-up of 2.9 years (IQR: 1.9–3.5), 26 subjects discontinued the treatment [10 due to toxicity, 7 for interactions with other drugs, 3 due to cardiovascular risk concern, 2 due to single viral blip, 1 due to VF, 1 for asthma, 1 patient’s decision, 1 due to enrolment in a study protocol].

**Conclusions:**

In this retrospective study, long-term use of RPV plus ABC/3TC regimen is effective and safe. Efficacy of this regimen was not found to be affected by low CD4+ nadir or high pre-ART viral load.

## Introduction

The efficacy and safety of an antiretroviral (ART) regimen based on rilpivirine/tenofovir/ emtricitabine (RPV/TDF/FTC) has been demonstrated in previous clinical trials [1–5].

RPV is currently licensed for use in combination with other antiretroviral agents, as a single agent or a single-tablet regimen with TDF and FTC, in antiretroviral-naïve and experienced patients, HIV-1-infected adults with <100.000 HIV-1 RNA copies/ml [4–7].

However, there is a need of additional data in regard to the use of RPV with other drugs, since in the trials above mentioned, few patients received RPV with the combination of abacavir/lamivudine (ABC/3TC), and it is not known if virological efficacy associated with a regimen based on RPV plus ABC/3TC might differ according to CD4+ nadir or pre-ART viral load. Indeed, these data could be useful in the current clinical management of patients, especially taking into account the well-known kidney and bone toxicity due to TDF [8,9].

The efficacy of a RPV plus ABC/3TC regimen in treatment-experienced patients was investigated in some previous retrospective studies showing 48-week proportions of virological efficacy ranging from 82% to 91% [10–12]. Moreover, the effectiveness and safety of the association of ABC/3TC plus RPV was also shown in treatment-naive HIV-1 patients in a recent study [13].

Therefore, the main purpose of this study was to assess the long-term efficacy of the switch to RPV plus ABC/3TC in virologically suppressed patients. The secondary objectives were to assess the safety of this regimen and whether the main study outcome is influenced by nadir CD4+ and pre-ART viral load.

## Methods

This is a retrospective, monocentric study on HIV-1 infected patients followed at the Infectious Disease Department of the San Raffaele Scientific Institute, HBsAg-negative, HLA B5701-negative, with HIV-RNA<50 copies/mL who started RPV plus ABC/3TC from March 2013 to September 2015. Patients with either (i) previously documented resistance (in historical resistance tests) to RPV, ABC or 3TC, or (ii) history of virological failure to rilpivirine, abacavir or lamivudine, were excluded from the study. The study protocol was approved by the Ethic Committee of San Raffaele hospital and all the enrolled patients provided written informed consent.

Efficacy was assessed in terms of durability and the primary outcome was the absence of treatment failure. The secondary outcomes were the virological failure and the associated resistance profile, changes in immunological and metabolic parameters and the safety profile associated with the study regimen.

Treatment failure (TF) was defined as the occurrence of virological failure (VF, 2 consecutive values >50 copies/mL) or discontinuation of any drug in the regimen for any reason.

Patients’ follow-up accrued from the date of RPV plus ABC/3TC initiation (baseline, BL) to the date of TF (VF or discontinuation for any cause of any drug in the regimen) or to the date of last available visit.

An a priori sample size evaluation estimated that 100 patients allowed to assess a 95% confidence interval not wider than 0.08 (i.e. 8%) around a treatment efficacy proportion ≥80% at 12- or 24-month follow-up.

Time to TF was evaluated by use of the Kaplan-Meier curves; TF was analysed in strata of nadir CD4+ (≤200 vs >200 cells/μl), pre ART viral load (<100000 vs ≥100000 copies/mL) and type of previous antiretroviral regimen [1 Protease Inhibitor (PI) + 2 Nucleoside Reverse Transcriptase Inhibitors (NRTIs), 1 Non-Nucleoside Reverse Transcriptase Inhibitors (NNRTI) + 2NRTIs, Other].

Predictors of TF were evaluated by a multivariate Cox regression model; the model included age, gender, HCV-Ab, nadir CD4+, viral load pre-ART, years of ART, years with undetectable VL at the start of the RPV plus ABC/3TC, backbone included in the previous regimen, previous antiretroviral regimen, baseline haemoglobin, baseline CD4+ and estimated glomerular factor rate (eGFR).

Mixed linear models were applied to evaluate changes in immunological, metabolic and other safety parameters.

## Results and discussion

Among 120 patients who started RPV plus ABC/3TC, 6 patients denied consent to the use of data in the analysis; 14 of the remaining 114 patients were excluded from this analysis as they started the study regimen with HIV RNA> 50 copies/ ml.

The median age was 48.7 (42.6–53.7) years, 80% were male, 79% Ab-anti HCV negative. The median years since HIV diagnosis was 11.0 (6.0–17.1), with a median nadir CD4+ 271 cells/μL (188–402). The median time on ART was 7.5 (4.4–14.1) years; 41 patients switched from a regimen based on PI+2NRTIs, 40 from 1 NNRTI+2NRTIs regimen and 19 from other treatments. In the majority of patients the backbone regimen was based on ABC/3TC (57%). The main reason for switching was the presence of toxicity (73%); among the 29 patients previously treated with a TDF-including regimen, 25 (86%) switched for renal toxicity. Patients’ characteristics are shown in Table 1 (more details are provided in S1–S4 Datasets).

**Table 1:** Patients’ characteristics at the start of the RPV plus ABC/3TC regimen.

Characteristic	Median (IQR) or n (%)
Age (years)	48.7 (42.6–53.7)
Male gender	80 (80%)
Body mass index (kg/m^2^)	23.8 (22.1–26.2)
• HIV risk factor	
• **IDU**	7 (7%)
• **MSM**	43 (43%)
• **Heterosex**	26 (26%)
• **Other/Unknown**	24 (24%)
Calendar year of start of the RPV plus ABC/3TC	2014 (2013–2014)
Years since HIV diagnosis	11.0 (6.0–17.1)
Years of antiretroviral therapy	7.5 (4.4–14.1)
Nadir CD4+ (cells/μL)	271 (188–402)
• **<200**	27 (27%)
• **≥200**	69 (69%)
• **Not available**	2 (2%)
Viral load pre-ART (log_10_copies/mL)	4.9 (4.4–5.4)
• **<100000**	42 (42%)
• **≥100000**	34 (34%)
• **Not available**	24 (24%)
Previous diagnosis of AIDS	10 (11%)
• Ab-anti HCV	
• **Negative**	79 (79%)
• **Positive**	17 (17%)
**Not available**	4 (4%)
Years with undetectable VL at the start of the RPV plus ABC/3TC	4.4 (1.8–6.4)
Previous antiretroviral regimen	
• **1 PI+2 NRTIs**	41 (41%)
• **1 NNRTI+2 NRTIs**	40 (40%)
• **Other**	19 (19%)
Antiretroviral drugs in use in the previous regimen	
• **Lamivudine**	64 (64%)
• **Emtricitabine**	30 (30%)
• **Abacavir**	57 (57%)
• **Zidovudine**	8 (8%)
• **Didanosine**	3 (3%)
• **Tenofovir**	29 (29%)
• **Efavirenz**	25 (25%)
• **Etravirine**	1 (1%)
• **Nevirapine**	5 (5%)
• **Rilpivirine**	10 (10%)
• **Atazanavir**	19 (19%)
• **Atazanavir/r**	11 (11%)
• **Darunavir/r**	10 (10%)
• **Lopinavir/r**	8 (8%)
• **Raltegravir**	4 (4%)
Most frequent (>5%) previous antiretroviral regimens	
• **Atazanavir+ abacavir+ lamivudine**	16 (16%)
• **Efavirenz+ tenofovir+emtricitabine**	13 (13%)
• **Efavirenz+ abacavir+lamivudine**	11 (11%)
• **Rilpivirine+tenofovir +emtricitabine**	9 (9%)
• **Atazanavir/r+ abacavir+ lamivudine**	7 (7%)
• **Zidovudine+abacavir+lamivudine**	6 (6%)
• **Darunavir/r+ abacavir+ lamivudine**	6 (6%)
Backbone included in the previous regimen	
• **Abacavir+lamivudine**	57 (57%)
• **Tenofovir+emtricitabine**	29 (29%)
• **Other**	14 (14%)
Reasons to switch to RPV plus ABC/3TC	
• **Simplification**	21 (21%)
• **Toxicity**	73 (73%)
• **Other/Not available**	6 (6%)

Abbreviations: ABC/3TC = abacavir/lamivudine, IDU = injection drug use, MSM = men who have sex with men, HCV = hepatits C virus, PI = protease inhibitor, NRTI = nucleoside reverse transcriptase inhibitors, NNRTI = Non Nucleoside Reverse Transcriptase Inhibitors.

**Fig 1:**
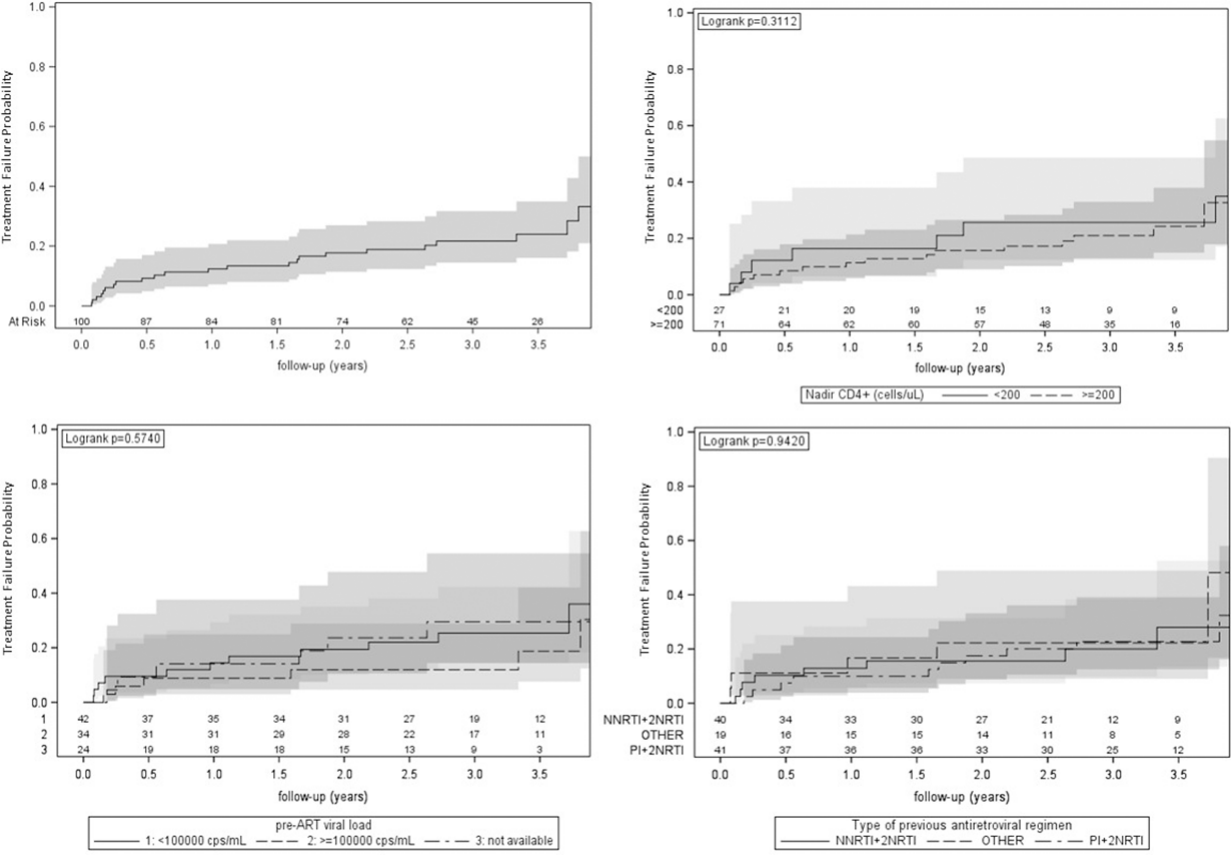
Treatment failure probability.

By 12, 24 and 36 months after switching to RPV plus ABC/3TC, the proportions of individuals without TF were 88% (95% CI:79%-93%), 82% (95% CI:73%-89%) and 78% (95% CI:68%-86%) respectively (Fig 1).

The time to TF was not significantly influenced by CD4+ cells nadir (≤200 vs >200 cells/μl; log-rank test: p = 0.311), viral load pre ART (<100000 vs ≥100000 copies/mL; log-rank test: p = 0.574) or the type of previous antiretroviral regimen (PI+2NRTIs vs NNRTI+2NRTIs vs Other; log-rank test: p = 0.942).

Over a median follow-up of 2.9 years (IQR: 1.9–3.5), 26 subjects discontinued the RPV plus ABC/3TC treatment; the most common reasons of discontinuation were toxicity (n = 10) [2 liver, 1 gastro-intestinal, 1 muscoloskeletal, 1 lipodystrophy, 5 dyslipidaemia (in 4 patients dyslipidemia persisted even after the discontinuation of this regimen)] and interactions with other drugs [use proton-pump inhibitors (n = 7)]. Reasons for discontinuation are shown in the Table 2.

**Table 2:** Reasons for discontinuation among subjects with undetectable viral load.

Reason for discontinuation	Duration of RPV+ ABC/3TC regimen (years)	Previous antiretroviral regimen	Pre-ART viral load (copies/mL)	Nadir CD4+ (cells/μL)	Baseline CD4+ (cells/μL)	End follow-up CD4+ (cells/μL)	End follow-up viral load (copies/mL)	ART regimen after discontinuation
Asthma	0.08	ATV/r+3TC	5500	260	691	671	<50	RAL+ETR
Concern of cardiovascular disease	3.91	EFV+FTC+TDF	73000	24	583	719	<50	RPV+FTC+TAF
Concern of cardiovascular disease	4.06	ATV+ABC+3TC	35902	333	670	622	<50	RPV+FTC+TAF
Concern of cardiovascular disease	3.81	ATV/r+ABC+3TC	391325	173	582	530	<50	RPV+FTC+TAF
Drug interaction	1.87	ATV+ABC+3TC	Not available	118	455	480	<50	RAL+ABC+3TC
Drug interaction	1.59	DRV/r+TDF+FTC	105100	508	608	1096	<50	DTG+ABC+3TC
Drug interaction	1.67	ATV/r+ABC+3TC	48000	117	497	632	<50	DTG+ABC+3TC
Drug interaction	3.33	EFV+ABC+3TC	278169	560	946	980	<50	DTG+ABC+3TC
Drug interaction	4.08	ATV/r+ABC+3TC	399100	63	442	886	<50	DTG+3TC
Drug interaction	0.17	RPV+FTC+TDF	22820	24	134	218	<50	DRV/r+FTC+TDF
Drug interaction	2.19	DRV/r+ABC+3TC	48310	269	538	623	<50	EVG+FTC+TDF+cobicistat
Dyslipidaemia	0.46	ATV+ABC+3TC	167078	329	541	830	<50	RAL+ABC+3TC
Dyslipidaemia	1.65	AZT+ABC+3TC	Not available	673	1344	1333	<50	DTG+ABC+3TC
Dyslipidaemia	0.11	EFV+FTC+TDF	87576	202	498	473	<50	EFV+FTC+TDF
Dyslipidaemia	3.72	DRV/r	20290	401	676	710	<50	DTG+3TC
Dyslipidaemia	0.08	ATV+FTC	46516	79	537	595	<50	ATV+3TC
Enrollment in a study protocol	0.64	RPV+3TC+TDF	270	396	1068	1080	<50	Experimental regimen
Lipodystrophy	0.97	AZT+ABC+3TC	27000	235	868	746	<50	DTG+ABC+3TC
Liver Toxicity	0.18	LPV/r+DDI+3TC	Not available	210	413	515	<50	RAL+3TC+ABC
Liver Toxicity	0.15	EFV+ABC+3TC	300000	853	1021	1030	<50	DTG+ABC+3TC
Patient’s wish	0.26	NVP+ABC+3TC	Not available	563	918	733	<50	NVP+ABC+3TC
Toxicity G-I tract	0.25	LPV/r+FTC+TDF	240000	77	231	243	<50	DRV/r+ABC+3TC
Toxicity, musculoskeletal	2.63	EFV+FTC+TDF	Not available	891	1140	1520	<50	DTG+3TC
Viral blip	2.72	ATV/r+ABC+3TC	85300	375	1178	1792	70	DTG+ABC+3TC
Viral blip	1.12	NVP+ABC+3TC	78370	382	790	988	93	DTG+ABC+3TC
Virological failure	0.56	ATV+ABC+3TC	Not available	156	1364	1180	21681	DRV/r+RAL+MVC

Abbreviations: ATV = atazanavir, ATV/r = atazanavir/ritonavir, ABC = abacavir, 3TC = lamivudine, DRV/r = darunavir/ritonavir, RAL = raltegravir, MVC = maraviroc, NVP = nevirapine, DTG = dolutegravir, LPV/r = lopinavir/ritonavir, FTC = emitricitabina, TDF = tenofovir, DDI = didanosine, ETR = etravirine, EFV = efavirenz, EVG+cobicistat = elvitegravir+cobicistat.

The only patient experiencing VF reached 21681 copies/mL with 1180 CD4 cells/μL six months after the introduction of RPV plus ABC/3TC; the resistance mutations detected at virological failure were: L100IL, K103N, E138AE. Two patients discontinued for viral blips: the first patient had a blip (89 copies/ml) 5 months after the introduction of the study regimen, followed by HIVRNA<50 copies/ml and then by a second blip (93 copies/ml) 10 months later; the second one had one blip (79 copies/ml) 6 months after the switch, followed by a two-year period with HIVRNA<50 copies/ml, and a second blip (70 copies/ml). The physicians didn’t ascribed the occurrence of viral blips to a lack of adherence.

A multivariate analysis assessing the predictive factors of treatment failure showed that none of the considered covariates was statistically associated with treatment failure.

Nine drug-related adverse events (AE) emerged during the study in 9 patients: 2 events of grade 1 (1 mild hepatotoxicity, 1 epigastralgia), 6 events of grade 2 (4 epigastralgia, 1 lipodystrophy, 1 arthralgia) and 1 event of grade 3 (arthralgia). The most common AE was epigastralgia (5/9 events); among subjects with epigastralgia (n = 5), 1 patient was treated for *Helicobacter pylori* infection and another was known being affected by gastroesophageal reflux disease (GERD).

Only one patient reported a serious adverse event (arthralgia) which resolved in the 6 months following the suspension of the study regimen.

Trend of immunological, metabolic and other safety parameters is reported in Table 3. A significant amelioration of CD4+ cells [32 (95% CI, 15–49), p = 0.0003), platelets [4.9 (2.2–7.6), p = 0.0005], alkaline phosphatase [-4.9 (-6.9, -3.0), p<0.0001] and serum calcium [+0.02 (0.01–0.04), p = 0.0001] was observed. A significant worsening of renal function was also found [serum creatinine; 0.04 (+0.03, +0.05), p<0.0001; eGFR with CKD-EPI formula (mL/min/1.73m2): -3.48 (-4.26, -2.69), p<0.0001] as well as a marginally significant increase in fasting insulin [0.87 (-0.09, +1.83), p = 0.074] not accompanied by a significant change in fasting glucose (p = 0.496) or HOMA-IR (p = 0.232).

**Table 3:** Univariate mixed linear models: Annual changes in immunological, metabolic and other safety parameters while on treatment with a RPV plus ABC/3TC regimen.

Characteristic	Baseline value	Crude change per year [Crude slope (95% CI)]		p-value
CD4+ (cells/μL)	691 (513–899)		+32 (+15, +49)	0.0003
Hb (g/dL)	14.9 (14.0–15.8)		+0.07 (+0.02, +0.13)	0.014
PLT (109/L)	216 (173–250)		+4.9 (+2.2, +7.6)	0.0005
AST (UI/L)	25 (19–33)		+0.16 (-1.6, +1.9)	0.858
ALT (UI/L)	31 (21–42)		-1.9 (-4.8, +1.0)	0.205
ALP (UI/L)	81 (66–100)		-4.9 (-6.9, -3.0)	<0.0001
FIB-4 index	1.02 (0.76–1.32)		+0.05 (-0.02, +0.12)	0.160
Total bilirubin (mg/dL)	0.52 (0.35–1.22)		-0.09 (-0.24, +0.05)	0.210
Direct bilirubin (mg/dL)	0.16 (0.11–0.29)		-0.03 (-0.07, +0.01)	0.194
Creatinine (mg/dL)	0.84 (0.71–1.01)		+0.04 (+0.03, +0.05)	<0.0001
eGFR with CKD-EPI formula (mL/min/1.73m2)	101 (85–110)		-3.48 (-4.26, -2.69)	<0.0001
Fasting glucose (mg/dL)	87 (80–93)		+0.9 (-1.7, +3.4)	0.496
Fasting insulin (mU/L)	7.3 (4.7–9.9)		+0.87 (-0.09, +1.83)	0.074
HOMA-IR	1.59 (1.00–2.21)		+0.19 (-0.12, +0.50)	0.232
Total cholesterol (mg/dL)	188 (169–220)		-0.12 (-3.5, +3.2)	0.944
LDL-cholesterol (mg/dL)	107 (95–125)		+0.4 (-1.8, +2.6)	0.725
HDL-cholesterol (mg/dL)	46 (40–55)		+0.4 (-0.5, +1.2)	0.398
Triglycerides (mg/dL)	117 (76–203)		+1.0 (-10.0, +12.1)	0.857
Calcium (mmol/L)	2.3 (2.2–2.4)		+0.02 (+0.01,+0.04)	0.0001
Phosphorous (mmol/L)	1.0 (0.9–1.1)		-0.002 (-0.02, +0.02)	0.862
Urinary protein (mg/dL)	5 (0–10)		-0.16 (-3.16, +2.84)	0.916

Abbreviations: Hb = hemoglobin, PLT = platelets, AST = aspartate aminotransferase, ALT = alanine aminotransferase, ALP = alkaline phosphatase, eGFR with CKD-EPI = estimated glomerular factor rate with Chronic Kidney Disease Epidemiology Collaboration, FIB-4 = fibrosis 4 calculator, HOMA-IR = insulin resistance index, LDL = low-density lipoprotein, HDL = high-density lipoprotein.

In this study we found a 88%, 82% and 78% efficacy at 12, 24 and 36 months after switching to RPV plus ABC/3TC with no significant differences in relation to CD4+ cells nadir or viral load pre ART or the type of previous antiretroviral regimen and thus supporting the use of this regimen even in patients with low CD4+ cells nadir or high viral load pre ART and regardless of the type of previous antiretroviral regimen.

Previous studies mainly reported 48-week efficacy results with the exception of the study by Marshall et al. [10]. The 48-week efficacy of our study is in agreement with those reported in controlled clinical studies [1–3] or in previous retrospective studies in treatment-experienced HIV-1 infected subjects switching to RPV plus ABC/3TC with undetectable viral load [10–13]. Marshall and colleagues published some data about the outcomes of 268 patients switching to RPV plus ABC/3TC as combination in fixed dose or to RPV/TDF/FTC [10]. This study reported similar virological efficacy: at 24 and 48 weeks of follow up (by an intention-to-treat analysis with switch = failure, missing = excluded), 87% and 82% of patients on RPV/ABC/3TC vs 77% and 73% of patients on RPV/TDF/FTC had viral load < 50 copies/ml; the 48-week results by the on-treatment analysis showed that 98% vs. 95% had viral load <50 copies/ml; the authors also showed a Kaplan-Meier curve with treatment discontinuation estimates up to week 108 which was approximately 23% at this time-point. The authors concluded that switching to ABC/3TC was as effective as the combination at fixed dose of RPV/FTC/TDF in maintaining virological suppression.

Recently, Palacios and colleagues reported about the efficacy and safety of switching to ABC/3TC plus RPV in virologically suppressed HIV infected patients on HAART [11]. At 48 weeks, 78 patients (92%) were still on treatment with this same regimen and the efficacy was 88% and 96% by the intention to treat and the per protocol analyses, respectively.

In the SIMRIKI study, at week 48, HIV RNA<50 copies/mL was maintained by 91% of patients by intention to treat analysis [12].

Our data also confirm the safety and good tolerability of the study regimen, already seen in registrative studies, i.e. ECHO and THRIVE [1–3] or in previous retrospective studies [10–13].

A significant improvement of CD4+ values with an increment of 32 cells/μL (95% confidence interval: +15, +49), haemoglobin, platelets, serum calcium and alkaline phosphatase was observed during the treatment with RPV plus ABC/3TC as well as a small and significant, although not clinically relevant, worsening of the renal function. The increase in CD4+ cell counts is likely the effect of the maintained virological suppression, while changes in bilirubin and in alkaline phosphatase are likely the effect of, respectively, atazanavir and tenofovir withdrawal. The reduction eGFR was expected, as it is known that RPV increases serum creatinine by inhibiting the OCT2 tubular transporter, which results in reduced tubular secretion of creatinine [14]. We do not have an obvious explanation for the favourable increase in haemoglobin levels, but we previously described similar findings in patients switching from PIs to RPV, FTC and TDF [15]. The use of RPV had no impact on lipids, liver function, phosphorous and urinary protein.

Based on these data, the RPV plus ABC/3TC regimen could be considered among treatment options for switch regimens due to its efficacy (in our study 1% of VF) also in patients with high pre-ART viral load or low nadir CD4+ cell count in addition to its safety and good tolerability. Over a median follow-up of approximately 3 years, only 10 patients changed the RPV plus ABC/3TC regimen for toxicity and only 7 patients for drug-drug interaction with proton-pump inhibitors. In fact, the RPV plus ABC/3TC regimen is characterised by a favourable drug-drug interaction profile. All these findings suggest that this regimen could be considered a good treatment option for patients with multimorbidity and the consequent problem of polypharmacy.

On the other hand, the limitations of this regimen include: the presence of ABC/3TC which precludes its use in patients with HLA B5701, its low genetic barrier and also the fact that it is not a single tablet regimen. In addition, caution in the use of the investigated regimen is also needed in patients with a high cardiovascular risk because of the presence of ABC/3TC.

The novelty of our findings relies on the long-term evaluation of the study regimen efficacy and safety and on the assessment of the influence of CD4+ cells nadir or viral load pre ART on these outcomes.

This study has some limitations that deserve discussion. The main one is the retrospective observational study design; clinicians may have prescribed this type of regimen at individuals with specific clinical characteristics at starting which may have somewhat limited the generalizability of the study results; however, this study included both subjects with low CD4 nadir (≤200 cells/μl) or high pre-ART viral load (≥100000 copies/mL) not always included in randomized trials that allowed the calculation of stratified analyses on these factors. Another important limitation is the lack of a control arm which can make it difficult to compare the effects observed under RPV plus ABC/3TC regimen with those of other ART regimens. The other limitations include the small number of patients in the strata of CD4+ nadir and pre-ART viral load and the lack of information on the anti-inflammatory properties of the considered drugs (no data on inflammation markers).

## Conclusions

In summary, data from long-term observation of this retrospective study, confirm the efficacy, safety and the good tolerability of the RPV plus ABC/3TC regimen. The efficacy of this regimen was not found to be affected by low CD4+ nadir or high pre-ART viral load.

## Supporting information

S1 Dataset Patients' clinical events. (PDF)

S2 Dataset Concomitant medications. (PDF)

S3 Dataset Demographical and clinical data. (PDF)

S4 Dataset Laboratory data. (PDF)
