# Supplementary material for: Long-term efficacy and safety of rilpivirine plus abacavir and lamivudine in HIV-1 infected patients with undetectable viral load
Source: PLoS One. 2018 Feb 16;13(2):e0191300. doi: 10.1371/journal.pone.0191300 (PMC5815573; doi:10.1371/journal.pone.0191300)
Supplement: S1 Dataset — (PDF) [file pone.0191300.s001.pdf]

ID	EVENT
1161	OROPHARYNGEAL CANDIDIASIS
1161	ESOPHAGEAL CANDIDIASIS
1161	LYPODYSTROPHY
1204	LYPODYSTROPHY
1234	OROPHARYNGEAL CANDIDIASIS
1234	KIDNEY DISEASE
1234	URINARY TRACT INFECTION
1234	ESOPHAGEAL CANDIDIASIS
1234	KIDNEY DISEASE
1234	HEADACHE, BONE PAIN,TUBULOPATHY
1304	SEBORRHEIC DERMATITIS
1304	SKIN DISEASE
1622	HEPATOMEGALY
1622	KIDNEY DISEASE
1675	HEPATOMEGALY
1675	HYPERTENSION BLOOD PRESSURE
2075	CMV PNEUMONIA
2075	PNEUMOCISTOSIS PNEUMONIA
2075	SEPSIS
2075	BACTERIAL PNEUMONIA
2075	ALLERGY TO DRUGS
2075	OROPHARYNGEAL CANDIDIASIS
2161	INGUINAL HERNIA
2161	SEBORRHEIC DERMATITIS
2161	OROPHARYNGEAL CANDIDIASIS
2161	HAIRY LEUCOPLAKIA
2484	KIDNEY STONE
2641	CONDYLOMATOSIS
2641	GYNECOMASTIA
2641	DIABETES
2641	ALLERGY TO DRUGS
2641	HYPERTENSION BLOOD PRESSURE
2890	LYPODYSTROPHY
2890	SKIN DISEASE
2912	HERPES SIMPLEX (SKIN)
2912	LYPODYSTROPHY
2912	INGUINAL HERNIA
2933	OSTEOPOROSIS
3203	HYPERTENSION BLOOD PRESSURE
3203	GYNECOMASTIA
3536	OROPHARYNGEAL CANDIDIASIS
3574	MOLLUSCUM CONTAGIOSUM
3574	MOLLUSCUM CONTAGIOSUM
3741	HERPES ZOSTER
3741	HERPES SIMPLEX OROPHARYNGEAL
3897	ALLERGY TO DRUGS
3897	OTITIS
3897	HEPATOMEGALY
3897	CONDYLOMATOSIS

3897	CONDYLOMATOSIS
3897	LYPODYSTROPHY
3897	SKIN CANDIDA INFECTION
3897	LYPODYSTROPHY
4016	HODGKIN LYNFOMA
4016	CONDYLOMATOSIS
4016	CONDYLOMATOSIS
4016	CONDYLOMATOSIS
4016	CONDYLOMATOSIS
4018	HERPES SIMPLEX OROPHARYNGEAL
4054	EXTRAPULMONARY TUBERCULOSIS
4054	HERPES SIMPLEX GENITAL
4054	LYPODYSTROPHY
4267	CRYPTOCOCCOSIS
4267	ESOPHAGEAL CANDIDIASIS
4267	CONDYLOMATOSIS
4267	HYPERTRIGLYCERIDEMIA
4282	PNEUMOCISTOSIS PNEUMONIA
4282	ALLERGY TO DRUGS
4282	ALOPECIA
4282	SKIN DISEASE
4668	TONSILLITIS
4668	DEPRESSION
4669	SKIN DISEASE
4669	SEBORRHEIC DERMATITIS
4669	SKIN DISEASE
4669	EXANTHEMA
4669	ALLERGY TO DRUGS
4669	SKIN DISEASE
4669	SKIN DISEASE
4974	DIABETES
5070	SYPHILIS
5192	PARATHYROID ADENOMA
5192	HEMORRHOIDS
5247	GENITAL CANDIDA
5288	ESOPHAGEAL CANDIDIASIS
5288	CHORIORETINITIS CMV INFECTION
5288	BACTERIAL PNEUMONIA
5288	PNEUMOCISTOSIS PNEUMONIA
5336	CONDYLOMATOSIS
5336	NON GONOCOCCAL URETHRITIS
5378	CONJUNCTIVITIS
5495	HERPES SIMPLEX (SKIN)
5495	HERPES SIMPLEX OROPHARYNGEAL
5687	CEREBRAL TOXOPLASMOSIS
5687	CEREBRAL TOXOPLASMOSIS
5687	CRIPTOCOCCOSIS
5687	URINARY TRACT INFECTION
5868	SYPHILIS
5868	HERPES SIMPLEX GENITAL

5868	INSULIN RESISTANCE
5868	HCV HEPATITIS
6222	PSYCHIATRIC DISORDERS
6222	HERPES ZOSTER
6307	PNEUMOCISTOSIS PNEUMONIA
6307	VISCERALE LEISHMANIOSIS
6307	BACTERIAL PNEUMONIA
6307	OROPHARYNGEAL CANDIDIASIS
6307	OSTEOPOROSIS
6461	BACTERIAL PNEUMONIA
6461	HERPES ZOSTER
6461	HERPES SIMPLEX (SKIN)
6461	SKIN DISEASE
6680	GONORRHEA INFECTION
6769	HERPES ZOSTER
6881	PSORIASIS
7200	PROCTITIS
7245	HODGKIN LYNFOMA
7283	ESOPHAGEAL CANDIDIASIS
7283	PNEUMOCISTOSIS PNEUMONIA
7756	SYPHILIS
7756	NON GONOCOCCAL URETHRITIS
7756	PROCTITIS
7756	PROCTITIS
8248	KIDNEY DISEASE
8248	PINWORMS
8490	HEADACHE
8824	CASTELMAN'S DISEASE
8833	LIVER CIRRHOSIS
8833	EXTRAPULMONARY TUBERCULOSIS
8833	LIVER CIRRHOSIS
8833	OSTEOPOROSIS
8855	SKIN INFECTION
8863	OTHER
8863	HPV INFECTION
8873	GLOMERULONEPHRITIS
8873	EYE DISORDERS
8965	KIDNEY DISEASE
8965	OSTEOPOROSIS
8965	KIDNEY DISEASE
9005	PROCTITIS
9005	HERPES SIMPLEX (SKIN)
9005	HCV HEPATITIS
9156	CONDYLOMATOSIS
9156	CONDYLOMATOSIS
9156	OTHER INFECTION
9156	CHRONIC BRONCHITIS
9424	EPILEPSY
9502	SYPHILIS
9587	BACTERIAL PNEUMONIA

9587	ENCEPHALITIS
9587	CMV SYSTEMIC INFECTION
9587	CMV SYSTEMIC INFECTION
9587	ACUTE HEPATITS
