# Supplementary material for: Long-term efficacy and safety of rilpivirine plus abacavir and lamivudine in HIV-1 infected patients with undetectable viral load
Source: PLoS One. 2018 Feb 16;13(2):e0191300. doi: 10.1371/journal.pone.0191300 (PMC5815573; doi:10.1371/journal.pone.0191300)
Supplement: S2 Dataset — (PDF) [file pone.0191300.s002.pdf]

ID	DRUG	POSOLOGY
864	FERRO-GRAD C	1 tab q24h
1161	AMARYL 3MG	1 tab q24h
1161	CARDIOASPIRIN 100MG	1 tab q24h
1161	DIBASE 25000UI/2,5ML	20 drops/week
1161	ESAPENT 1G	q8h
1161	GLICLAZIDE 80MG	1 tab q24h
1161	LANTUS	q24h
1161	LEVEMIR	q12h
1161	LOSAPREX 50MG	1 tab q24h
1161	METFORMIN 500MG	q8h
1161	NOVONORM 0,5MG	1 tab q24h
1161	NOVORAPID	q8h
1161	OLANZAPINE 10 MG	1 tab q24h
1161	PRAVASELECT 40 MG	1/2tab q24h
1161	TRESIBA	q24h
1161	VENLAFAX. BLF 75MG	2 tabs q12h
1161	ZARELIS 150MG	1 tab q24h
1234	AIRCORT SPRAY 50MCG	dosage variable
1234	ALENDRONATE 70MG	1 tab/week
1234	ARCOXIA 60MG	1 tab q24h
1234	ARIANNA	1 tab q24h
1234	BENERVA 100MG/1ML	1 phial every other day
1234	CALC CAR EG 1G	1 tab q24h
1234	CRESTOR 10MG	1 tab q24h
1234	PREDNISONE 25MG	1/2tab q24h
1234	PREDNISONE 5MG	1/2tab q24h
1234	DEURSIL-RR MITE 225MG	1 tab q24h
1234	DIBASE 10000UI/ML 10ML	20 drops/week
1234	DOMPERIDONE 10MG	1 tab q24h
1234	DOMPERIDONE 10MG	1 tab q24h
1234	EN 1 MG	1 tab q24h
1234	ESOMEPRAZOLO 40MG	1 tab q24h
1234	FAXINE 75MG	1 tab q24h
1234	FOSTER 100/6MCG	q12h
1234	FUROSEM 25MG	1 tab q24h
1234	IMOVANE 7,5MG	1 tab q24h
1234	KESTINE 10MG	1 tab q24h
1234	LAMICTAL 50MG	1 tab q24h
1234	LAROXYL	q24h
1234	LUCEN 40MG	2 tabs q12h
1234	LYRICA 150MG	2 tabs q12h
1234	LYRICA 150MG	1 tab q24h
1234	NOOTROPIL 1200MG	2 tabs q12h
1234	PLAQUENIL 200MG	1 tab q24h
1234	RIVOTRI 2 MG	1 tab q24h
1234	TARGIN 10MG/5MG	2 tabs q12h
1234	TRITTICO 50 MG	1 tab q24h
1234	VENTOLIN	q12h
1250	DIBASE 10000UI/ML 10ML	30 drops/week

1340	CORTONE ACETATE 25 MG	q12h
1340	CORTONE ACETATE 25 MG	q8h
1340	CRESTOR 5MG	1 tab q24h
1340	EUTIROX 100MCG	1 tab q24h
1340	EUTIROX 125MCG	1 tab q24h
1340	LEXOTAN 2,5MG/ML	dosage variable
1622	CRESTOR 10MG	1 tab q24h
1622	DIBASE 10000UI/ML 10ML	20 drops/week
1622	OMNIC 0,4MG	1 tab q24h
1675	CRESTOR 5MG	1 tab q24h
1675	TALAVIR 1000MG	q8h
2194	BENADON 300MG	1 tab q24h
2194	DIBASE 10000UI/ML 10ML	20 drops/week
2194	FOLINIC ACID 5 MG	1 tab q24h
2194	MODURETIC 5MG/50MG	1/2tab q24h
2194	RAMIPRIL ABC 5MG	1 tab q24h
2194	RAMIPRIL ABC 5MG	1/2tab q24h
2194	RAMIPRIL ABC 5MG	1 tab q24h
2393	ZYLORIC 300MG.	1 tab q24h
2484	CACIT VIT.D3 1G/880UI	1 tab q24h (Monday to Friday)
2484	CRESTOR 10MG	1 tab q24h
2484	CRESTOR 20MG	1 tab q24h
2484	CRESTOR 20MG	1 tab q24h
2484	DIBASE 25000UI/2,5ML	20 drops/week
2484	METOCAL VIT.D3	15days/month
2641	APIDRA 100U/ML 3ML	q8h
2641	DIAMICRON 30MG	2 tabs q24h
2641	DIBASE 10000UI/ML 10ML	30 drops/week
2641	FENOFIBRATE200MG	1 tab q24h
2641	LANTUS	q8h
2641	LOBIVON 5 MG	1/2tab q24h
2641	NORVASC 5MG	1/2tab q24h
2641	RAMIPRIL AGE 10MG	1 tab q24h
2641	SIMESTAT 5MG	1 tab q24h
2912	CRESTOR 10MG	1 tab q24h
2912	DIBASE 10000UI/ML 10ML	20 drops/week
2933	DIBASE 25000UI/2,5ML	1 phial/month
3203	CARDIOASPIRIN 100MG	1 tab q24h
3203	DEURSIL 150MG	1 tab q24h
3203	DIBASE 10000UI/ML 10ML	30 drops/week
3203	FULCRO*200MG 20 CPS	1 tab q24h
3203	KARVEA 150MG	1 tab q24h
3203	LIBRADIN 10MG	1 tab q24h
3203	NATRILIX 2,5MG	1 tab q24h
3203	ZYLORIC 300MG.	1/2tab q24h
3536	PEGASYS 180MCG	1 phial/week
3536	REBETOL 200 MG	2 tabs q12h
3574	CARDIOASPIRIN 100MG	1 tab q24h
3574	CRESTOR 10MG	1 tab q24h

3574	HIROBRIZ BREEZ. 150MCG	1 tab q24h
3713	ANGELIQ 2MG	1 tab q24h
3713	DIBASE 10000UI/ML 10ML	20 drops/week
3713	FILENA	1 tab q24h
3741	CRESTOR 10MG	1 tab q24h
3741	DIBASE 10000UI/ML 10ML	20 drops/week
3824	METFORMIN 500MG	1 tab q24h
3824	METFORMIN 500MG	1 tab q12h
3897	CRESTOR 10MG	1 tab q24h
3897	CRESTOR 10MG	1 tab q24h
3897	DIBASE 10000UI/ML 10ML	20 drops/week
3897	FENOFIBRATE200MG	1 tab q24h
4018	DIBASE 10000UI/ML 10ML	20 drops/week
4018	PRAVASTAT.ACC 20MG	1 tab q24h
4054	DIBASE 10000UI/ML 10ML	20 drops/week
4054	LEXOTAN 2,5MG/ML	dosage variable
4054	RISEDRONIC ACID 35MG	1 tab/week
4172	CRESTOR 10MG	1 tab q24h
4172	DIBASE 10000UI/ML 10ML	20 drops/week
4172	RAMIPRIL ABC 5MG	1 tab q24h
4267	LORTAAN 12,5MG	1 tab q24h
4279	ATORVASTATIN 10MG	1 tab q24h
4282	DIDROGYL*1,5MG/10ML	[illegible] drops/day for 20 day each [illegible]
4282	OPTINATE 35MG	1 tab/week
4282	OROTRE 500MG+400UI	2 tabs q24h
4282	RISEDRONIC ACID 35MG	1 tab/week
4669	ABILIFY 10MG	1 tab q24h
4669	ABILIFY 10MG	1 tab q24h
4669	DEPAKIN 1000MG	1 tab q24h
4669	DIBASE 10000UI/ML 10ML	25 drops/week
4669	EN 1 MG	dosage variable
4669	FARLUTAL 10 MG	1 tab q24h every 15 days
4669	FENOFIBRATE200MG	1 tab q24h
4669	OLANZAPINE 10 MG	1 tab q24h
4669	PRAVASTAT.ACC 20MG	1 tab q24h
4669	SERTRALINE 100MG	1 tab q24h
4669	SERTRALINE 100MG	1 tab q24h
5192	CALCIUM SANDOZ 1000MG	q8h
5192	COAPROVEL 150/12,5 MG	1/2tab q24h
5192	CRESTOR 20MG	1 tab q24h
5192	CRESTOR 40MG	1 tab q24h
5192	DIBASE 10000UI/ML 10ML	25 drops/week
5192	PRAVASELECT 40 MG	1 tab q24h
5192	ROCALTROL 0,25MCG	q8h
5192	ZIRTEC 10MG	1 tab q24h
5247	CYMBALTA 60MG	1 tab q24h
5247	DIBASE 10000UI/ML 10ML	20 drops/week
5247	PAROXETINE 20MG	1 tab q24h
5247	PREFOLIC 15 MG	1 tab q24h
5247	PRIMOLUT NOR 10MG	1 tab q24h

5288	DIBASE 10000UI/ML 10ML	1 phial/month
5288	ESKIM 1000 MG	1 tab q12h
5288	FULCRO 200MG	1 tab q24h
5378	CRESTOR 10MG	1 tab q24h
5495	DIBASE 100000UI/ML 6F	1 phial/month
5495	PEGASYS 180MCG	1 phial/week
5495	REBETOL 200 MG	1 tab q12h
5495	XANAX 0,25MG	1 tab q8h
5571	ANTRA 20 MG	1 tab q24h
5571	CALC CAR EG 1G	1 tab q24h
5571	CRESTOR 5MG	1 tab q24h
5571	DIBASE 10000UI/ML 10ML	30 drops/week
5571	ESKIM 1000 MG	1 tab q24h
5571	ESOPRAL 40MG	1 tab q24h
5571	FULCROSUPRA 145MG	1 tab q24h
5571	GAVISCON 200 ML	dosage variable
5571	GEMFIBROZIL SAN 900MG	q8h
5571	MOTILIUM 10MG	q8h
5687	NATECAL D3 600MG+400UI	2 tabs q24h
5782	RAMIPRIL ABC 5MG	1 tab q24h
5868	ANSIOLIN 5MG	dosage variable
5868	BENZIL 1200000UI	2 phial week, for 3 weeks
5868	CRESTOR 10MG	1 tab q24h
5868	CRESTOR 5MG	1 tab q24h
5868	DIBASE 10000UI/ML 10ML	20 drops/week
5868	DISIPAL 50MG	1 tab q24h
5868	FULCRO 200MG	1 tab q24h
5868	SEREUPIN 20MG	1 tab q24h
5868	ZYPREXA 2,5 MG	1 tab q24h
6009	FULCRO 200MG	1 tab q24h
6034	DIBASE 25000UI/2,5ML	30 drops/week
6060	AKINETON 4MG	1 tab q24h
6060	ANDROCUR 100MG	1 tab q12h
6060	BISOPROLOL 1,25MG	1 tab q24h
6060	CIPRALEX 20MG/ML	q24h
6060	DEPAKIN CHRONO 500 MG	1 tab q12h
6060	DIBASE 10000UI/ML 10ML	30 drops/week
6060	ETHINYLESTRADIOL 50MCG	1 tab q24h
6060	HALDOL 30 ML 1%	q12h
6060	SEROQUEL 100MG	q8h
6222	ABILIFY 10MG	1 tab q24h
6222	CARDIOASPIRIN 100MG	1 tab q24h
6222	ISOPTIN 40MG	1 tab q12h
6222	LORAZEPAM 1MG	1/2tab q24h
6222	PAROXETINE 20MG	1 tab q24h
6222	PAROXETINE 20MG	1/2tab q24h
6222	RYTMONORM 325MG	1 tab q12h
6222	TAVOR 1MG	1/2tab q24h
6307	ALIFLUS DISKUS 50/500	1 puff q12h

6307	DIBASE 10000UI/ML 10ML	25 drops/week
6307	DIBASE 10000UI/ML 10ML	5 drops q24h
6307	GABAPENTIN ABC 300MG	dosage variable
6307	GABAPENTIN ABC 300MG	q8h
6307	PARACETAMOLO 500MG	dosage variable
6461	MESALAZINA 800MG	q8h
6461	PERINDOPRIL 4MG	1 tab q24h
6672	AUGMENTIN 875MG+125MG	1 tab q12h
6672	DIBASE 10000UI/ML 10ML	25 drops/week
6680	ANTRA 20 MG	1 tab q24h
6680	DIBASE 10000UI/ML 10ML	30 drops/week
6680	KLAVOX 875+125	q8h
6751	AC ACSAL MOR 100MG	1 tab q24h.
6751	AMLODIPINE 5MG	1/2tab q24h
6751	DIBASE 25000UI/2,5ML	20 drops/week
6751	ENALAPRIL ALM 5MG	1 tab q24h
6751	KARVEZIDE 150/12,5 MG	1 tab q24h
6751	KARVEZIDE 300/25MG	1 tab q24h
6751	TOTALIP 10 MG	1 tab every other day
6769	CRESTOR 40MG	1 tab q24h
6769	DIBASE 10000UI/ML 10ML	20 drops/week
6769	FULCRO 200MG	1 tab q24h
6790	CARDURA 2MG	1 tab q24h.
6790	CARDURA 2MG	1 tab q24h.
6790	CRESTOR 10MG	1 tab q24h.
6790	FULCRO 200MG	1 tab q24h.
6790	LASIX 25MG	1/2tab q24h
6790	LOBIVON 5 MG	1/2tab q24h
6790	METFORMIN 500MG	1 tab q12h
6790	TIKLID 250MG	1 tab q12h
6790	TRIATEC 5MG	1 tab q24h.
6881	CALCIUM D3 SANDOZ	1 tab q24h.
6881	CRESTOR 10MG	1 tab q24h.
6881	CRESTOR 5MG	1 tab q24h.
6881	DIBASE 100000UI/ML 6F	1 phial/month
6881	DIBASE 10000UI/ML 10ML	30 drops/week
6881	DIBASE 25000UI/2,5ML	1 phial/month
6881	ESKIM 1000 MG	1 tab q12h
6881	ESKIM 1000 MG	1 tab q12h
6881	METFORMIN 500MG	1 tab q24h.
6881	NEOTIGASON 10MG	2 tabs q24h
6944	CRESTOR 10MG	1 tab q24h.
6944	DISSENTEN 2MG	1 tab q24h.
6944	FULCRO 200MG	1 tab q24h.
6953	DIBASE 10000UI/ML 10ML	20 drops/week
6955	VIAGRA 25 MG	1 tab q24h.
6972	DIBASE 10000UI/ML 10ML	20 drops/week
6972	DIBASE 25000UI/2,5ML	20 drops/week
6972	TAVANIC 500MG	1 tab q24h.
7137	DIBASE 10000UI/ML 10ML	30 drops/week

7145	DIBASE 25000UI/2,5ML	1 phial/month
7200	ANTROLIN*0,3%+1,5% 30 G	q12h
7200	DIBASE 10000UI/ML 10ML	30 drops/week
7200	DIBASE 10000UI/ML 10ML	30 drops/week
7283	DIBASE 10000UI/ML 10ML	20 drops/week
7283	FULCRO 200MG	1 tab q24h.
7283	FULCRO 200MG	1 tab q24h.
7283	FULCRO 200MG	1 tab q24h.
7408	BIFAZOL*1% 30 G	q12h
7408	CORTIFLUORAL 10ML	1 tab q24h
7756	DIBASE 10000UI/ML 10ML	20 drops/week
7756	RISEDRONIC ACID 35MG	1 tab q24h.
7853	DIBASE 10000UI/ML 10ML	30 drops/week
7853	LANTUS	dosage variable
7853	NOVORAPID	dosage variable
7918	BACTRIM 800/160 MG	1 tab q24h.
7918	DIBASE 10000UI/ML 10ML	30 drops/week
7935	DROPAXIN*10MG/ML 30ML	q24h
7935	EN 1MG/ML 20ML	q24h
7935	IDROCLOR FN 25MG	q8h
7935	METOCAL 1250MG	1 tab q24h.
8063	CRESTOR 10MG	1 tab q24h
8063	CRESTOR 10MG	1 tab q24h
8063	DIBASE 25000UI/2,5ML	1 phial/month
8063	IDEOS	1 tab q24h.
8063	RANITIDINE AGE 300MG	1 tab q24h.
8103	APROVEL 150 MG	1 tab q24h.
8103	DIBASE 10000UI/ML 10ML	25 drops/week
8103	INDERAL 40MG	1/2tab q24h
8103	METFORMIN 500MG	1 tab q24h.
8103	PROVISACOR 20M	1 tab q24h.
8103	REPAGLINIDE EG 1MG	1 tab q12h
8118	DIBASE 100000UI/ML 6F	1 phial/month
8118	DIBASE 25000UI/2,5ML	1 phial/month
8153	DIBASE 25000UI/2,5ML	1 phial/month
8248	FULCRO 200MG	1 tab q24h.
8328	CEFIXORAL 400 MG	1 tab q24h (Monday to Friday)
8328	CLENIL 0,8MG/2ML	q12h
8328	FLUIMUCIL 300MG/3ML	q12h
8334	DIBASE 10000UI/ML 10ML	30 drops/week
8334	DIBASE 25000UI/2,5ML	1 phial/month
8490	CRESTOR 10MG	1 tab q24h.
8490	CRESTOR 10MG	1 tab q24h.
8490	ESKIM 1000 MG	1 tab q24h.
8490	LAMISIL125 MG	1 tab q24h.
8490	MICARDIS 20MG	1 tab q24h.
8575	CEFIXORAL 400 MG	1 tab q24h.
8797	DIBASE 10000UI/ML 10ML	25 drops/week
8824	ALFUZOSIN 10MG	1 tab q24h.

8824	AUGMENTIN 875MG+125MG	q8h
8824	CARDIOASPIRIN 100MG	1 tab q24h.
8824	CIALIS 20MG	dosage variable
8824	CRESTOR 10MG	1 tab q24h.
8824	DIBASE 10000UI/ML 10ML	30 drops/week
8824	KLACID 500MG	1 tab q12h
8824	PANTORC 40MG	1 tab q24h.
8824	PENTACARINAT 300MG	q24h
8824	PLAVIX 75MG	1 tab q24h.
8824	RANITIDINE AGE 300MG	1 tab q24h.
8824	VELAMOX 1G	q8h
8824	VIAGRA 25 MG	dosage variable
8833	BENADON 300MG	1 tab q24h.
8833	BENERVA 300MG	1 tab q24h.
8833	CALCIUM SANDOZ 1000MG	1 bag q24h.
8833	CARDIOASPIRIN 100MG	1 tab q24h
8833	CONTRAMAL 200MG	1 tab q12h
8833	DIDROGYL*1,5MG/10ML	20 drops/week
8833	ETAPIAM 500 MG	q8h
8833	KANRENOL 100 MG	1 tab q24h.
8833	KCL-RETARD 600 MG	1 tab q24h.
8833	LAEVOLAC EPS SCIR. 180 ML	q8h
8833	LASIX 25MG	1 tab q12h
8833	NICOZID 200 MG	2 tabs q12h
8833	PYRAZINAMIDE 500MG	3tabs q24h
8833	RIFADIN 600MG	1 tab q24h.
8855	AUGMENTIN 875MG+125MG	q8h
8855	DIBASE 10000UI/ML 10ML	30 drops/week
8855	VIAGRA 25 MG	dosage variable
8863	BASSADO 100 MG	1 tab q12h
8863	DIBASE 100000UI/ML 6F	1 phial/month
8873	DIBASE 100000UI/ML 6F	1 phial/month
8873	DIBASE 100000UI/ML 6F	1phial each 2 months
8873	FULCROSUPRA 145MG	1 tab q24h.
8873	FULCROSUPRA 145MG	1 tab q24h.
8873	NEBIVOLOL 5MG	1/2tab q24h
8873	RAMIPRIL ABC 5MG	1 tab q24h.
8873	RAMIPRIL FG 2,5MG	1 tab q24h.
8965	DIBASE 10000UI/ML 10ML	40 drops/week
8965	RISEDRONIC ACID 35MG	1 tab q24h.
9005	CARBOLITHIUM 300MG	3tabs q24h
9005	PAROXETINE 20MG	1 tab q24h.
9081	CRESTOR 10MG	1 tab q24h.
9081	DIBASE 10000UI/ML 10ML	30 drops/week
9395	DIBASE 25000UI/2,5ML	1 phial/month
9395	DIBASE 25000UI/2,5ML	1 phial/month
9395	DIBASE 25000UI/2,5ML	1 phial/month
9395	DIBASE 25000UI/2,5ML	1 phial/month
9424	DIBASE 10000UI/ML 10ML	20 drops/week
9424	TOLEP 300MG	q8h

9452	CONTRAMAL 100MG/ML	dosage variable
9452	CRESTOR 10MG	1 tab q24h.
9452	DIDROGYL*1,5MG/10ML	4 drops q24h/3weeks each months
9452	RISEDRONIC ACID 35MG	1 tab/week
9452	SERACTIL 400MG	dosage variable
9456	DIBASE 10000UI/ML 10ML	20 drops/week
9479	DIBASE 10000UI/ML 10ML	25 drops/week
9483	CIALIS 10MG	dosage variable
9483	DIBASE 10000UI/ML 10ML	25 drops/week
9483	METFORMIN 500MG	1 tab q24h.
9483	METFORMIN 500MG	1 tab q12h
9483	PLACTIDIL 300 MG	1 tab q24h.
9483	PLAVIX 75MG	1 tab q24h.
9502	DIBASE 25000UI/2,5ML	1 phial/month
9502	DIBASE 25000UI/2,5ML	1 phial/month
9561	DIBASE 100000UI/ML 6F	1 phial/month
9587	ATENOLOL 00MG 42tab	1/4tab q24h
9587	CARDIOASPIRIN 100MG	1 tab q24h.
9587	DIBASE 100000UI/ML 6F	25 drops/week
9587	DIBASE 10000UI/ML 10ML	20 drops/week
9587	FULCROSUPRA 145MG	1 tab q24h.
9587	LANTUS	q12h
9587	LASIX 25MG	1 tab q24h.
9587	METFORMIN 500MG	q8h
9587	MICARDIS 80MG	1 tab q24h
9587	MICARDIS PLUS 40/12,5MG	1 tab q24h.
9587	NOVORAPID	q8h
9637	DIBASE 10000UI/ML 10ML	40 drops/week
9682	OMNIC 0,4MG	1 tab q24h.
9901	DIBASE 10000UI/ML 10ML	20 drops/week
