# Supplementary material for: Long-term efficacy and safety of rilpivirine plus abacavir and lamivudine in HIV-1 infected patients with undetectable viral load
Source: PLoS One. 2018 Feb 16;13(2):e0191300. doi: 10.1371/journal.pone.0191300 (PMC5815573; doi:10.1371/journal.pone.0191300)
Supplement: S3 Dataset — (PDF) [file pone.0191300.s003.pdf]

ID	AGE	AGE_UPD	YEARS_HIV	YEARS_HIV_UPD	HIV RISK FACTOR	NAIVE	HIVRNA_PREART	YEARS_ARV	YEARS_ARV_UPD	NADIR_CD4	HCV	HBV	CDC	TIME_FU	TIME_FU_UPD	TIME_SOP	TC	ABC	ATV	AZT	DDI	DRV	EFV	ETV	FTC	LPV	NVP	RAL	RPV	RTV	TDF	NRTI	NNRTI	PI	II	UNKNOWN	REASON	ARV_POST	VIREMIA_BL	VIREMIA	BIP	FAIL	VIREMIA_CHANGE_BL	HIVDNA_BL	HIVDNA	HIVDNA_CHANGE_BL	CD4_BL	CD4	CD4_CHANGE_BL	CD8_BL	CD8	CD8_CHANGE_BL	CD4P_BL	CD4P	CD4P_CHANGE_BL	CD8P_BL	CD8P	CD8P_CHANGE_BL	CDRAPP_BL	CDRAPP	CDRAPP_CHANGE_BL	Hb_BL	Hb	Hb_CHANGE_BL	PLT_BL	PLT	PLT_CHANGE_BL	SGOT_BL	SGOT	SGOT_CHANGE_BL	SGPT_BL	SGPT	SGPT_CHANGE_BL	ALP_BL	ALP	ALP_CHANGE_BL	BILTOT_BL	BILTOT	BILTOT_CHANGE_BL	BILDIR_BL	BILDIR	BILDIR_CHANGE_BL	CREA_BL	CREA	CREA_CHANGE_BL	GLU_BL	GLU	GLU_CHANGE_BL	CHOLEST_BL	CHOLEST	CHOLEST_CHANGE_BL	EGFR_BL	EGFR	EGFR_CHANGE_BL	BILIND_BL	BILIND	BILIND_CHANGE_BL	CALCIUM_BL	CALCIUM	CALCIUM_CHANGE_BL	PHOSPHOR_BL	PHOSPHOR	PHOSPHOR_CHANGE_BL	COLLDL_BL	COLLDL	COLLDL_CHANGE_BL	COLHDL_BL	COLHDL	COLHDL_CHANGE_BL	TRIG_BL	TRIG	TRIG_CHANGE_BL	FIB_BL	FIB	FIB_CHANGE_BL	U_PRO_BL	U_PRO
0	42,0616016	42,1437372	21,6673511	21,74948665	HOMOSEXUAL	0	999	17,412731	17,49486653	156	0	9	B3	0,55852156	0,082135524	6,20396988	1	1	1	0	0	0	0	0	0	0	0	0	0	0	0	1	0	1	0	0	Virological failure	DRV/r+RAL+MVC	0,9	36	99	99	35,1				1364	957	-407													16,5	16	-0,8	234	243	9	34	25	-9	41	34	-7	109	92	-17	4,27	0,81	-3,46	1,07	0,2	-0,87	0,84	0,88	0,04	88	76	-12	187	176	-11	108	106	-2	3,2	0,61	-2,59	2,46	2,25	-0,21	0,7	0,61	-0,09	100	107	7	41	40	-1	192	209	17	0,95	0,7	-0,21	9	0
864	50,4284736	50,4284736	28,9746749	28,97467488	HETEROSEXUAL	0	29907	5,66735113	5,667351129	276	9	0	A1	2,45037645	0	0,20533881	0	0	0	0	0	0	0	0	0	0	0	1	1	0	0	0	1	0	1	0			0,9		1		0				765		0	592			45			26,9			1,76			15		0	223		0	24		0	31		0										0,75		0	82		0	231		0	93		0																66		0	0,97		0		
864	50,4284736	50,587269	28,9746749	29,13347023	HETEROSEXUAL	0	29907	5,66735113	5,826146475	276	9	0	A1	2,45037645	0,158795346	0,20533881	0	0	0	0	0	0	0	0	0	0	0	1	1	0	0	0	1	0	1	0			0,9	322	1		321,1				765	1031	266	592			45	49,1	4,1	26,9			1,76			15	15	0,2	223	216	-7	24	16	-8	31	15	-16										0,75	0,62	-0,13	82	78	-4	231	236	5	93	105	12																66	80	14	0,97	1	0,01		
864	50,4284736	50,770705	28,9746749	29,31690623	HETEROSEXUAL	0	29907	5,66735113	6,009582478	276	9	0	A1	2,45037645	0,342231348	0,20533881	0	0	0	0	0	0	0	0	0	0	0	1	1	0	0	0	1	0	1	0			0,9	0,9			0				765			592			45			26,9			1,76			15			223			24			31												0,75			82			231			93																		66			0,97				
864	50,4284736	51,192334	28,9746749	29,73853525	HETEROSEXUAL	0	29907	5,66735113	6,431211499	276	9	0	A1	2,45037645	0,76386037	0,20533881	0	0	0	0	0	0	0	0	0	0	0	1	1	0	0	0	1	0	1	0			0,9	39			38,1				765	1005	240	592			45	45,7	0,7	26,9			1,76			15	15	0,1	223	219	-4	24	17	-7	31	17	-14										0,75	0,71	-0,04	82	78	-4	231	221	-10	93	99	6																66	116	50	0,97	1	-0,01		0
864	50,4284736	51,7563313	28,9746749	30,30253251	HETEROSEXUAL	0	29907	5,66735113	6,995208761	276	9	0	A1	2,45037645	1,327857632	0,20533881	0	0	0	0	0	0	0	0	0	0	0	1	1	0	0	0	1	0	1	0			0,9	0,9			0				765	836	71	592			45	44	-1	26,9			1,76			15	15	-0,2	223	237	14	24	20	-4	31	9	-22										0,75	0,73	-0,02	82	86	4	231	246	15	93	95	2											114			134		66	61	-5	0,97	1,5	0,49		
864	50,4284736	52,3613963	28,9746749	30,90759754	HETEROSEXUAL	0	29907	5,66735113	7,600273785	276	9	0	A1	2,45037645	1,932922656	0,20533881	0	0	0	0	0	0	0	0	0	0	0	1	1	0	0	0	1	0	1	0			0,9	0,9			0				765	998	233	592			45	47,5	2,5	26,9			1,76			15	15	-0,1	223	257	34	24	19	-5	31	19	-12										0,75	0,83	0,08	82	87	5	231	212	-19	93	81	-12											103			113		66	68	2	0,97	0,9	-0,09		
864	50,4284736	52,8788501	28,9746749	31,42505133	HETEROSEXUAL	0	29907	5,66735113	8,117727584	276	9	0	A1	2,45037645	2,450376454	0,20533881	0	0	0	0	0	0	0	0	0	0	0	1	1	0	0	0	1	0	1	0			0,9	39			38,1				765	991	226	592	502	-90	45	47,2	2,2	26,9	23,9	-3	1,76	1,97	0,21	15	16	0,5	223	235	12	24	22	-2	31	24	-7										0,75	0,78	0,03	82	85	3	231	249	18	93	87	-6																66	133	67	0,97	1	0,04		
[illegible]																																																																																																																														

5133	53,6563997	55,6221766	9,62628337	11,59206023	HETEROSEXUAL	0	173130	8,66255989	10,62833676	602	0	0	A1	4,20260096	1,965776865	8,31759069	1	1	0	0
5133	53,6563997	56,3066393	9,62628337	12,27652293	HETEROSEXUAL	0	173130	8,66255989	11,31279945	602	0	0	A1	4,20260096	2,650239562	8,31759069	1	1	0	0
5133	53,6563997	56,7857632	9,62628337	12,75564682	HETEROSEXUAL	0	173130	8,66255989	11,79192334	602	0	0	A1	4,20260096	3,12936345	8,31759069	1	1	0	0
5133	53,6563997	57,3415469	9,62628337	13,31143053	HETEROSEXUAL	0	173130	8,66255989	12,34770705	602	0	0	A1	4,20260096	3,685147159	8,31759069	1	1	0	0
5133	53,6563997	57,8590007	9,62628337	13,82888433	HETEROSEXUAL	0	173130	8,66255989	12,86516085	602	0	0	A1	4,20260096	4,202600958	8,31759069	1	1	0	0
5184	48,5475702	48,5475702	9,79055441	9,790554415	HOMOSEXUAL	0	1023933	9,76865161	9,768651608	315	0	0	B2	3,69883641	0	0	0	0	0	0
5184	48,5475702	48,9089665	9,79055441	10,15195072	HOMOSEXUAL	0	1023933	9,76865161	10,13004791	315	0	0	B2	3,69883641	0,361396304	0	0	0	0	0
5184	48,5475702	48,91718	9,79055441	10,16016427	HOMOSEXUAL	0	1023933	9,76865161	10,13826146	315	0	0	B2	3,69883641	0,369609856	0	0	0	0	0
5184	48,5475702	48,9746749	9,79055441	10,21765914	HOMOSEXUAL	0	1023933	9,76865161	10,19575633	315	0	0	B2	3,69883641	0,427104723	0	0	0	0	0
5184	48,5475702	49,1279945	9,79055441	10,37097878	HOMOSEXUAL	0	1023933	9,76865161	10,34907598	315	0	0	B2	3,69883641	0,580424367	0	0	0	0	0
5184	48,5475702	49,2950034	9,79055441	10,53798768	HOMOSEXUAL	0	1023933	9,76865161	10,51608487	315	0	0	B2	3,69883641	0,747433265	0	0	0	0	0
5184	48,5475702	49,7713895	9,79055441	11,01437372	HOMOSEXUAL	0	1023933	9,76865161	10,99247091	315	0	0	B2	3,69883641	1,223819302	0	0	0	0	0
5184	48,5475702	50,2724162	9,79055441	11,51540041	HOMOSEXUAL	0	1023933	9,76865161	11,4934976	315	0	0	B2	3,69883641	1,724845996	0	0	0	0	0
5184	48,5475702	50,8090349	9,79055441	12,05201916	HOMOSEXUAL	0	1023933	9,76865161	12,03011636	315	0	0	B2	3,69883641	2,26146475	0	0	0	0	0
5184	48,5475702	51,247091	9,79055441	12,49007529	HOMOSEXUAL	0	1023933	9,76865161	12,46817248	315	0	0	B2	3,69883641	2,699520876	0	0	0	0	0
5184	48,5475702	51,8110883	9,79055441	13,05407255	HOMOSEXUAL	0	1023933	9,76865161	13,03216975	315	0	0	B2	3,69883641	3,263518138	0	0	0	0	0
5184	48,5475702	52,2464066	9,79055441	13,48939083	HOMOSEXUAL	0	1023933	9,76865161	13,46748802	315	0	0	B2	3,69883641	3,698836413	0	0	0	0	0
5192	55,9698836	55,9698836	8,99931554	8,999315537	HETEROSEXUAL	0	35902	8,03011636	8,030116359	333	0	0	A2	3,96714579	0	7,54825462	1	1	1	0
5192	55,9698836	56,1861739	8,99931554	9,215605749	HETEROSEXUAL	0	35902	8,03011636	8,246406571	333	0	0	A2	3,96714579	0,216290212	7,54825462	1	1	1	0
5192	55,9698836	56,3942505	8,99931554	9,423682409	HETEROSEXUAL	0	35902	8,03011636	8,454483231	333	0	0	A2	3,96714579	0,424366872	7,54825462	1	1	1	0
5192	55,9698836	56,5612594	8,99931554	9,590691307	HETEROSEXUAL	0	35902	8,03011636	8,621492129	333	0	0	A2	3,96714579	0,59137577	7,54825462	1	1	1	0
5192	55,9698836	56,6105407	8,99931554	9,639972621	HETEROSEXUAL	0	35902	8,03011636	8,670773443	333	0	0	A2	3,96714579	0,640657084	7,54825462	1	1	1	0
5192	55,9698836	57,1088296	8,99931554	10,13826146	HETEROSEXUAL	0	35902	8,03011636	9,169062286	333	0	0	A2	3,96714579	1,138945927	7,54825462	1	1	1	0
5192	55,9698836	57,6563997	8,99931554	10,68583162	HETEROSEXUAL	0	35902	8,03011636	9,716632444	333	0	0	A2	3,96714579	1,686516085	7,54825462	1	1	1	0
5192	55,9698836	57,6810404	8,99931554	10,71047228	HETEROSEXUAL	0	35902	8,03011636	9,741273101	333	0	0	A2	3,96714579	1,711156742	7,54825462	1	1	1	0
5192	55,9698836	57,8261465	8,99931554	10,85557837	HETEROSEXUAL	0	35902	8,03011636	9,886379192	333	0	0	A2	3,96714579	1,856262834	7,54825462	1	1	1	0
5192	55,9698836	58,1136208	8,99931554	11,1430527	HETEROSEXUAL	0	35902	8,03011636	10,17385352	333	0	0	A2	3,96714579	2,143737166	7,54825462	1	1	1	0
5192	55,9698836	58,1957563	8,99931554	11,22518823	HETEROSEXUAL	0	35902	8,03011636	10,25598905	333	0	0	A2	3,96714579	2,22587269	7,54825462	1	1	1	0
5192	55,9698836	58,4996578	8,99931554	11,52908966	HETEROSEXUAL	0	35902	8,03011636	10,55989049	333	0	0	A2	3,96714579	2,529774127	7,54825462	1	1	1	0
5192	55,9698836	58,7652293	8,99931554	11,79466119	HETEROSEXUAL	0	35902	8,03011636	10,82546201	333	0	0	A2	4,1615332	2,795345654	7,54825462	1	1	1	0
5192	55,9698836	58,9787817	8,99931554	12,00821355	HETEROSEXUAL	0	35902	8,03011636	11,03901437	333	0	0	A2	4,1615332	3,008898015	7,54825462	1	1	1	0
5192	55,9698836	59,7097878	8,99931554	12,73921971	HETEROSEXUAL	0	35902	8,03011636	11,77002053	333	0	0	A2	4,1615332	3,739904175	7,54825462	1	1	1	0
5192	55,9698836	59,7152635	8,99931554	12,74469541	HETEROSEXUAL	0	35902	8,03011636	11,77549624	333	0	0	A2	4,1615332	3,745379877	7,54825462	1	1	1	0
5192	55,9698836	59,9370294	8,99931554	12,96646133	HETEROSEXUAL	0	35902	8,03011636	11,99726215	333	0	0	A2	4,1615332	3,967145791	7,54825462	1	1	1	0

7756	43,08282	45,5359343	2,99247091	5,445585216	HOMOSEXUAL	0	12920	2,99247091	5,445585216	447	0	0	A2	3,43326489	2,453114305	0,50924025	0	0	0	0	0	0
7756	43,08282	45,6646133	2,99247091	5,574264203	HOMOSEXUAL	0	12920	2,99247091	5,574264203	447	0	0	A2	3,52908966	2,581793292	0,50924025	0	0	0	0	0	0
7756	43,08282	45,982204	2,99247091	5,891854894	HOMOSEXUAL	0	12920	2,99247091	5,891854894	447	0	0	A2	3,52908966	2,899383984	0,50924025	0	0	0	0	0	0
7756	43,08282	46,3463381	2,99247091	6,255989049	HOMOSEXUAL	0	12920	2,99247091	6,255989049	447	0	0	A2	3,52908966	3,263518138	0,50924025	0	0	0	0	0	0
7756	43,08282	46,45859	2,99247091	6,368240931	HOMOSEXUAL	0	12920	2,99247091	6,368240931	447	0	0	A2	3,52908966	3,375770021	0,50924025	0	0	0	0	0	0
7756	43,08282	46,5160849	2,99247091	6,425735797	HOMOSEXUAL	0	12920	2,99247091	6,425735797	447	0	0	A2	3,52908966	3,433264887	0,50924025	0	0	0	0	0	0
7853	36,6160164	36,6160164	5,63449692	5,63449692	HOMOSEXUAL	0	24383	5,0294319	5,029431896	321	0	0	A2	3,26351814	0	4,81587953	1	1	1	0	0	0
7853	36,6160164	36,8405202	5,63449692	5,859000684	HOMOSEXUAL	0	24383	5,0294319	5,253935661	321	0	0	A2	3,26351814	0,224503765	4,81587953	1	1	1	0	0	0
7853	36,6160164	37,0568104	5,63449692	6,075290897	HOMOSEXUAL	0	24383	5,0294319	5,470225873	321	0	0	A2	3,26351814	0,440793977	4,81587953	1	1	1	0	0	0
7853	36,6160164	37,1581109	5,63449692	6,176591376	HOMOSEXUAL	0	24383	5,0294319	5,571526352	321	0	0	A2	3,26351814	0,542094456	4,81587953	1	1	1	0	0	0
7853	36,6160164	37,3305955	5,63449692	6,349075975	HOMOSEXUAL	0	24383	5,0294319	5,744010951	321	0	0	A2	3,26351814	0,714579055	4,81587953	1	1	1	0	0	0
7853	36,6160164	37,5085558	5,63449692	6,527036277	HOMOSEXUAL	0	24383	5,0294319	5,921971253	321	0	0	A2	3,26351814	0,892539357	4,81587953	1	1	1	0	0	0
7853	36,6160164	37,5359343	5,63449692	6,554414784	HOMOSEXUAL	0	24383	5,0294319	5,94934976	321	0	0	A2	3,26351814	0,919917864	4,81587953	1	1	1	0	0	0
7853	36,6160164	37,8617385	5,63449692	6,880219028	HOMOSEXUAL	0	24383	5,0294319	6,275154004	321	0	0	A2	3,26351814	1,245722108	4,81587953	1	1	1	0	0	0
7853	36,6160164	37,8672142	5,63449692	6,88569473	HOMOSEXUAL	0	24383	5,0294319	6,280629706	321	0	0	A2	3,26351814	1,25119781	4,81587953	1	1	1	0	0	0
7853	36,6160164	37,9000684	5,63449692	6,918548939	HOMOSEXUAL	0	24383	5,0294319	6,313483915	321	0	0	A2	3,26351814	1,284052019	4,81587953	1	1	1	0	0	0
7853	36,6160164	38,255989	5,63449692	7,274469541	HOMOSEXUAL	0	24383	5,0294319	6,669404517	321	0	0	A2	3,26351814	1,639972621	4,81587953	1	1	1	0	0	0
7853	36,6160164	38,6255989	5,63449692	7,644079398	HOMOSEXUAL	0	24383	5,0294319	7,039014374	321	0	0	A2	3,26351814	2,009582478	4,81587953	1	1	1	0	0	0
7853	36,6160164	39,0417522	5,63449692	8,060232717	HOMOSEXUAL	0	24383	5,0294319	7,455167693	321	0	0	A2	3,26351814	2,425735797	4,81587953	1	1	1	0	0	0
7853	36,6160164	39,4661191	5,63449692	8,484599589	HOMOSEXUAL	0	24383	5,0294319	7,879534565	321	0	0	A2	3,26351814	2,850102669	4,81587953	1	1	1	0	0	0
7853	36,6160164	39,8795346	5,63449692	8,898015058	HOMOSEXUAL	0	24383	5,0294319	8,292950034	321	0	0	A2	3,26351814	3,263518138	4,81587953	1	1	1	0	0	0
7918	48,1998631	48,1998631	13,8151951	13,81519507	HETEROSEXUAL	0	70000	7,35660507	7,356605065	130	0	0	C3	1,63723477	0	6,40109514	0	0	0	0	0	0
7918	48,1998631	48,4681725	13,8151951	14,08350445	HETEROSEXUAL	0	70000	7,35660507	7,624914442	130	0	0	C3	1,63723477	0,268309377	6,40109514	0	0	0	0	0	0
7918	48,1998631	49,1143053	13,8151951	14,72963723	HETEROSEXUAL	0	70000	7,35660507	8,271047228	130	0	0	C3	1,63723477	0,914442163	6,40109514	0	0	0	0	0	0
7918	48,1998631	49,8370979	13,8151951	15,45242984	HETEROSEXUAL	0	70000	7,35660507	8,993839836	130	0	0	C3	1,63723477	1,637234771	6,40109514	0	0	0	0	0	0
7935	26,8856947	26,8856947	5,95756331	5,957563313	UNKNOWN	1	33441	0	0	475	0	0	B2	2,59548255	0	0	0	0	0	0	0	0
7935	26,8856947	27,0061602	5,95756331	6,078028747	UNKNOWN	1	33441	0	0,120465435	475	0	0	B2	2,59548255	0,120465435	0	0	0	0	0	0	0
7935	26,8856947	27,2772074	5,95756331	6,349075975	UNKNOWN	1	33441	0	0,391512663	475	0	0	B2	2,59548255	0,391512663	0	0	0	0	0	0	0
7935	26,8856947	27,890486	5,95756331	6,962354552	UNKNOWN	1	33441	0	1,004791239	475	0	0	B2	2,59548255	1,004791239	0	0	0	0	0	0	0
7935	26,8856947	28,3559206	5,95756331	7,427789185	UNKNOWN	1	33441	0	1,470225873	475	0	0	B2	2,59548255	1,470225873	0	0	0	0	0	0	0
7935	26,8856947	28,6625599	5,95756331	7,734428474	UNKNOWN	1	33441	0	1,776865161	475	0	0	B2	2,59548255	1,776865161	0	0	0	0	0	0	0
7935	26,8856947	29,0896646	5,95756331	8,161533196	UNKNOWN	1	33441	0	2,203969884	475	0	0	B2	2,59548255	2,203969884	0	0	0	0	0	0	0
7935	26,8856947	29,4811773	5,95756331	8,553045859	UNKNOWN	1	33441	0	2,595482546	475	0	0	B2	2,59548255	2,595482546	0	0	0	0	0	0	0
8063	38,844627	38,844627	4,7063655	4,706365503	HOMOSEXUAL	0	1258	1,41820671	1,418206708	418	0	0	A2	3,61396304	0	1,33059548	1	1	0	0	0	1

[illegible]

U_PRO_CHANGE_BL	U_HB_BL	U_HB	U_HB_CHANGE_BL	HEIGHT_BL	WEIGHT_BL	WEIGHT
9	9	0	9	174	67	62
		0				
0	0		0	173	64	64
	0			173	64	64
10	0	0	0	173	64	64
0	0	0	0	173	64	64
				173	64	63
0	0	0	0	173	64	63
5	0	0	0	173	64	63
				173	64	64
5	0	0	0	173	64	64
5	0	0	0	173	64	64
				173	64	62
10	0	0	0	173	64	
5	0	0	0	173	64	
				174	67	67
		0		174	67	62
		0,06		174	67	62
				174	67	62
0	0		0	150	42	42
9	0			150	42	62
170	0	0,06	0,06	150	42	62
20	0	0	0	150	42	62
				150	42	43
				170	69	69
		0		170	69	43
				170	69	73
		0,03		170	69	73
		0		170	69	73
				170	69	73
		0		170	69	75
		0		170	69	71
				170	69	78
0	0,06		0	175	82	82
	0,06			175	82	73
-10	0,06	0,06	0	175	82	73
-10	0,06	0,06	0	175	82	73
-10	0,06	0,03	-0,03	175	82	73
-10	0,06	0,06	0	175	82	73
0	0,06	0	-0,06	175	82	
-10	0,06	0	-0,06	175	82	
-10	0,06	0	-0,06	175	82	
-10	0,06	0	-0,06	175	82	
0	0		0			
	0					73
0	0	0	0			73
	0					73
0	0	0	0			73
	0					73
	0					73
	0					
	0					
	0					
	0					
	0					
	0					
0	0		0	183	75	75
-45	0	0	0	183	75	76
-40	0	0	0	183	75	76
-45	0	0	0	183	75	75
-50	0	0	0	183	75	75
-50	0	0	0	183	75	77
0	0		0	175	66	66
	0			175	66	75
0	0	0,03	0,03	175	66	75
5	0	0	0	175	66	69
5	0	0,03	0,03	175	66	70
5	0	0,03	0,03	175	66	67
0	0	0	0	175	66	67
10	0	0	0	175	66	68
0	0	0	0	175	66	72
0	0	0,03	0,03	175	66	72
						67
						67
						67
						67
						67
						67
						67
						67
				191	84	84
				191	84	67
				191	84	77
		0		191	84	77
		0		191	84	77
				191	84	77
				191	84	77
		0		191	84	78
		0		191	84	78
		0		191	84	78
		0		191	84	78
		0		191	84	78
		0		191	84	78
0	0		0	175	77	77
	0			175	77	77
	0			175	77	77
	0			175	77	77
	0			175	77	77
0	0	0	0	175	77	
	0			175	77	
0	0	0	0	175	77	
	0			175	77	
				174	74	74
				174	74	77
				174	74	74
				174	74	74
				174	74	74
				174	74	72
				174	74	73
				174	74	74
				174	74	74
				174	74	74
				174	74	74
0	0,03		0	175	68	68
	0,03			175	68	72
-50	0,03	0,03	0	175	68	72
-50	0,03	0,03	0	175	68	72
				175	68	68
-100	0,03	0,03	0	175	68	68
50	0,03	0,1	0,07	175	68	68
0	0,03	0,03	0	175	68	68
-80	0,03	0	-0,03	175	68	68
				175	68	70
0	0,03	0,03	0	175	68	70
-50	0,03	0,03	0	175	68	70
0	0,03	0,03	0	175	68	70
-50	0,03	0,03	0	175	68	70
-50	0,03	0,03	0	175	68	70
-80	0,03	0	-0,03	175	68	70
-50	0,03	0,03	0	175	68	71
-80	0,03	0	-0,03	175	68	71
-80	0,03	0,06	0,03	175	68	71
-80	0,03	0	-0,03	175	68	71
-100	0,03	0	-0,03	175	68	71
						70
						70
						70
		0				
0	0		0	176	70	70
	0			176	70	70
10	0	0	0	176	70	70
				176	70	70
10	0	0	0	176	70	70
				176	70	70
10	0	0	0	176	70	70
				176	70	69
10	0	0	0	176	70	69
0	0	0	0	176	70	69
				168	55	55
		0		168	55	69
		0		168	55	
				168	55	
		0		168	55	
				168	55	
0	0		0	180	90	90
5	0	0	0	180	90	69
5	0	0	0	180	90	69
0	0	0	0	180	90	69
				180	90	90
10	0	0	0	180	90	90
5	0	0	0	180	90	90
				180	90	91
10	0	0	0	180	90	91
0	0	0	0	180	90	89
0	0	0	0	180	90	89
20	0	0	0	180	90	89
0	0	0	0	180	90	89
0	0	0	0	180	90	80
0	0		0	174	56	56
	0			174	56	91
	0			174	56	91
0	0	0	0	174	56	91
				174	56	58
0	0		0	174	64	64
	0			174	64	58
0	0		0		70	70
	0				70	58
10	0	0	0		70	58
	0				70	
	0				70	
	0				70	
0	0		0	160	67	67
	0			160	67	58
0	0	0	0	160	67	58
	0			160	67	71
0	0		0	173	82	82
	0			173	82	71
	0			173	82	71
	0			173	82	71
60	0	0	0	173	82	71
0	0	0	0	173	82	71
-5	0	0	0	173	82	
	0			173	82	
-10	0	0	0	173	82	
				170	68	68
				170	68	71
				170	68	71
				170	68	72
				170	68	72
				170	68	68
						68
		0				
0	1		0			
	1					68
	1					68
	1					
	1					
0	0		0	172	78	78
	0			172	78	68
	0			172	78	68
0	0	0	0	172	78	68
				172	78	75
	0			172	78	75
0	0	0,03	0,03	172	78	75
				172	78	76
5	0	0,03	0,03	172	78	
0	0		0	149	48	48
	0			149	48	76
5	0	0	0	149	48	76
	0			149	48	76
	0			149	48	76
	0			149	48	76
-5	0	0	0	149	48	76
	0			149	48	50
-5	0	0	0	149	48	50
	0			149	48	50
0	0		0	150	50	50
0	0	0,1	0,1	150	50	76
	0			150	50	76
	0			150	50	76
0	0	0,06	0,06	150	50	76
	0			150	50	76
0	0	0,03	0,03	150	50	76
0	0	0,03	0,03	150	50	53
0	0	0	0	150	50	53
0	0	0	0	150	50	53
0	0		0	175	82	82
	0			175	82	76
-5	0	0	0	175	82	76
	0			175	82	76
				175	82	87
-5	0	0	0	175	82	87
-5	0	0	0	175	82	87
-5	0	0	0	175	82	87
	0			175	82	87
5	0	0	0	175	82	87
-5	0	0	0	175	82	87
-5	0	0	0	175	82	87
5	0	0	0	175	82	87
-5	0	0	0	175	82	87
	0			175	82	87
	0			175	82	87
-5	0	0	0	175	82	87
	0			175	82	87
-5	0	0	0	175	82	88
	0			175	82	85
5	0	0	0	175	82	85
0	0		0	170	69	69
-50	0	0	0	170	69	87
50	0	0	0	170	69	87
				170	69	65
-80	0	0	0	170	69	65
				170	69	70
50	0	0	0	170	69	70
				170	69	75
-50	0	0	0	170	69	74
-100	0	0	0	170	69	74
	0			170	69	74
	0			170	69	74
-150	0	0	0	170	69	74
	0			170	69	74
	0			170	69	74
	0			170	69	74
	0			170	69	74
	0			170	69	74
	0			170	69	74
-120	0	0	0	170	69	74
0	0		0	167	53	53
0	0	0,03	0,03	167	53	75
				167	53	55
0	0	0	0	167	53	55
	0			167	53	55
				167	53	48
				167	53	47
	0			167	53	47
0	0	0	0	167	53	47
-5	0	0,03	0,03	167	53	49
				178	72	72
		0		178	72	49
				178	72	74
				158	55	55
				158	55	74
				158	55	74
				158	55	74
				158	55	74
				158	55	74
		0		158	55	74
				158	55	65
		0		158	55	
				158	55	
				158	55	
				182	95	95
		0		182	95	65
		0		182	95	65
		0		182	95	65
				182	95	103
		0		182	95	103
		0		182	95	84
0	0		0	176	69	69
5	0	0	0	176	69	103
-5	0	0	0	176	69	70
-5	0	0	0	176	69	70
0	0,03		0	179	71	71
	0,03			179	71	103
-5	0,03	0	-0,03	179	71	103
	0,03			179	71	103

	0,03			179	71	103
	0,03			179	71	
	0,03			179	71	
	0,03			179	71	
	0,03			179	71	
0	0		0			
0	0	0	0			103
	0					103
	0					103
	0					103
	0					103
	0					103
	0					103
	0					
	0					
0	0	0	0			
	0					
0	0		0	175	83	83
	0			175	83	103
	0			175	83	103
20	0	0	0	175	83	103
				175	83	86
30	0	0	0	175	83	86
50	0	0	0	175	83	86
				175	83	86
30	0	0	0	175	83	86
30	0	0	0	175	83	86
				175	83	87
5	0	0	0	175	83	87
20	0	0	0	175	83	
50	0	0	0	175	83	
	0			175	83	
10	0	0	0	175	83	
30	0	0	0	175	83	
0	0		0	165	54	54
0	0	0	0	165	54	87
	0			165	54	87
				165	54	58
				172	63	63
				172	63	58
				172	63	58
				172	63	
				172	63	
				172	63	
				172	63	
				171	77	77
		0		171	77	58
				171	77	58
		0		171	77	58
				171	77	76
		0		171	77	76
				171	77	76
		0		171	77	76
		0		171	77	75
		0		171	77	75
		0		171	77	75
0	0		0	172	69	69
	0			172	69	76
	0			172	69	76
	0			172	69	76
	0			172	69	76
	0			172	69	76
-10	0	0	0	172	69	76
	0			172	69	76
	0			172	69	
	0			172	69	
	0			172	69	
				180	78	78
		0		180	78	76
		0		180	78	76
				180	78	76
		0		180	78	76
				180	78	84
		0		180	78	83
		0		180	78	83
		0		180	78	83
0	0		0	180	80	80
-5	0	0	0	180	80	84
	0			180	80	84
	0			180	80	84
-5	0	0	0	180	80	84
-5	0	0	0	180	80	84
	0			180	80	84
	0			180	80	84
-5	0	0	0	180	80	84
-5	0	0	0	180	80	84
				180	80	80
-5	0	0	0	180	80	80
				180	80	80
0	0	0	0	180	80	80
				180	80	79
	0			180	80	79
0	0	0	0	180	80	82
5	0	0	0	180	80	82
	0			180	80	82
	0			180	80	82
-5	0	0	0	180	80	82
	0			180	80	82
-5	0	0	0	180	80	82
	0			180	80	82
-5	0	0	0	180	80	82
-5	0	0	0	180	80	81
	0			180	80	81
-5	0	0	0	180	80	81
-5	0	0	0	180	80	81
0	0		0		70	70
0	0,06		0			
	0,06					79
	0,06					79
	0,06					79
	0,06					79
	0,06					
	0,06					
	0,06					
0	0		0	178	75	75
	0			178	75	79
				178	75	78
0	0	0	0	178	75	78
	0			178	75	78
0	0	0	0	178	75	82
	0			178	75	82
0	0	0	0	178	75	82
0	0	0	0	178	75	82
0	0,5		0	172	80	80
	0,5			172	80	78
	0,5			172	80	78
	0,5			172	80	78
	0,5			172	80	78
	0,5			172	80	78
	0,5			172	80	78
	0,5			172	80	
	0,5			172	80	
	0,5			172	80	
					66	66
					66	78
		0			66	78
0	0		0	173	94	94
	0			173	94	88
				162	65	65
				162	65	88
				162	65	88
				162	65	88
				162	65	
				169	81	81
		0		169	81	88
				169	81	88
				169	81	88
				169	81	88
				169	81	78
		0		169	81	78
		0		169	81	78
				169	81	78
		0		169	81	78
0	0,03		0	183	63	63
-40	0,03	0	-0,03	183	63	88
0	0		0	184		
	0			184		88
-5	0	0	0	184		88
-5	0	0	0	184		88
0	0	0	0	184		88
5	0	0	0	184		88
				184		82
-5	0	0	0	184		82
0	0		0	170	64	64
	0			170	64	82
5	0	0	0	170	64	82
0	0	0	0	170	64	82
				170	64	64
	0			170	64	64
	0			170	64	64
0	0	0	0	170	64	64
10	0	0	0	170	64	64
20	0	0	0	170	64	67
10	0	0	0	170	64	67
0	0	0	0	170	64	67
0	0		0	174	55	55
	0			174	55	64
	0			174	55	64
	0			174	55	64
				174	55	58
0	0	0	0	174	55	58
				174	55	57
5	0	0	0	174	55	59
0	0	0	0	174	55	61
0	0	0	0	174	55	61
				182	71	71
		0		182	71	57
		0		182	71	57
				182	71	57
		0		182	71	57
				182	71	
				182	71	
0	0		0	175	60	60
-5	0	0	0	175	60	57
	0			175	60	57
0	0	0	0	175	60	57
				175	60	69
-5	0	0	0	175	60	69
5	0	0	0	175	60	69
				175	60	65
	0			175	60	65
	0			175	60	65
	0			175	60	65
	0			175	60	65
0	0	0	0	175	60	65
	0			175	60	65
0	0	0	0	175	60	65
				175	60	67
	0			175	60	67
5	0	0	0	175	60	67
5	0	0	0	175	60	67
	0			175	60	67
0	0		0	175	78	78
0	0	0	0	175	78	67
	0			175	78	67
				175	78	77
0	0	0	0	175	78	78
10	0	0	0	175	78	78
0	0		0	173	71	71
10	0	0	0	173	71	77
	0			173	71	77
	0			173	71	77
	0			173	71	77
0	0	0	0	173	71	77
	0			173	71	
	0			173	71	
0	0,03		0	170	105	105
	0,03			170	105	77
50	0,03	0	-0,03	170	105	77
				170	105	109
0	0,03	0	-0,03	170	105	109
0	0,03	0	-0,03	170	105	109
50	0,03	0	-0,03	170	105	109
-30	0,03	0	-0,03	170	105	109
0	0,03	0	-0,03	170	105	111
-50	0,03	0	-0,03	170	105	111
-30	0,03	0	-0,03	170	105	108
-30	0,03	0	-0,03	170	105	108
0	0		0	168	66	66
5	0	0	0	168	66	109
	0			168	66	109
0	0		0	170	69	69
	0			170	69	109
	0			170	69	109
	0			170	69	109
-5	0	0	0	170	69	109
				170	69	70
-5	0	0	0	170	69	70
0	0	0	0	170	69	70
-5	0	0	0	170	69	71
	0			170	69	71
-5	0	0	0	170	69	71
-5	0	0	0	170	69	71
0	0		0	155	58	58
0	0	0	0	155	58	70
5	0	0	0	155	58	70
5	0	0	0	155	58	
	0			155	58	
5	0	0	0	155	58	
0	0		0	175	57	57
	0			175	57	70
0	0	0	0	175	57	70
				175	57	59
0	0		0	187	72	72
	0			187	72	59
	0			187	72	59
				187	72	72
	0			187	72	72
0	0	0,03	0,03	187	72	72
-5	0	0	0	187	72	72
	0			187	72	72
-5	0	0	0	187	72	70
-5	0	0	0	187	72	70
-5	0	0	0	187	72	70
0	0		0	170	72	72
	0			170	72	72
-5	0	0	0	170	72	72
				170	72	72
0	0	0	0	170	72	72
5	0	0	0	170	72	72
	0			170	72	73
0	0		0	170	66	66
0	0	0,2	0,2	170	66	72
	0			170	66	72
5	0	0	0	170	66	72
				170	66	65
0	0	0	0	170	66	65
				170	66	64
0	0	0	0	170	66	64
				170	66	66
0	0	0	0	170	66	66
5	0	0	0	170	66	66
0	0		0	180	74	74
	0			180	74	66
	0			180	74	66
-10	0	0	0	180	74	66
	0			180	74	66
	0			180	74	
	0			180	74	
0	0		0	181	75	75
	0			181	75	66
	0			181	75	66
	0			181	75	66
	0			181	75	66
	0			181	75	
	0			181	75	
0	0		0	180	80	80
0	0	0	0	180	80	66
0	0	0	0	180	80	66
	0			180	80	66
	0			180	80	66
0	0	0	0	180	80	66
	0			180	80	66
0	0	0	0	180	80	66
0	0	0	0	180	80	
	0			180	80	
0	0	0	0	180	80	
	0			180	80	
0	0	0	0	180	80	
	0			180	80	
	0			180	80	
0	0	0	0	180	80	
0	0	0	0	180	80	
0	0		0	185	86	86
0	0	0	0	185	86	66
0	0		0	170	63	63
5	0	0	0	170	63	66
				165	54	54
		0		165	54	66
				165	54	66
				165	54	66
				165	54	66
		0		165	54	66
				165	54	53
				165	54	53
				165	54	53
				165	54	53
				165	54	54

		0		165	54	54
				165	54	54
				165	54	54
				165	54	54
				165	54	54
				165	54	54
				189	76	76
		0		189	76	54
				189	76	78
		0		189	76	78
		0		189	76	78
		0		189	76	78
				189	76	78
		0		189	76	78
				189	76	78
				189	76	79
		0		189	76	78
		0		189	76	81
		0		189	76	80
		0		189	76	83
		0		189	76	84
0	0		0	174	86	86
-15	0	0	0	174	86	
-20	0	0	0	174	86	
0	0	0	0	174	86	
0	0		0	160	56	56
	0			160	56	79
	0			160	56	79
	0			160	56	79
	0			160	56	
	0			160	56	
0	0	0	0	160	56	
	0			160	56	
				175	79	79
				175	79	79
				175	79	77
				175	79	77
				175	79	78
				175	79	78
				175	79	78
				175	79	78
				175	79	78
				175	79	77
				175	79	75
				175	79	76
0	0,03		0	179	88	88
	0,03			179	88	78
-5	0,03	0	-0,03	179	88	78
	0,03			179	88	78
	0,03			179	88	78
	0,03			179	88	78
	0,03			179	88	78
0	0,2		0	183	92	92
30	0,2	0,2	0	183	92	78
130	0,2	0,5	0,3	183	92	78
				183	92	94
0	0,2	0,2	0	183	92	94
180	0,2	0,5	0,3	183	92	
230	0,2	0,2	0	183	92	
0	0,03		0	173	72	72
	0,03			173	72	94
	0,03			173	72	94
-5	0,03	0	-0,03	173	72	94
				173	72	70
-10	0,03	0	-0,03	173	72	
	0,03			173	72	
	0,03			173	72	
0	0		0	175	73	73
5	0	0	0	175	73	73
5	0	0	0	175	73	73
0	0	0	0	175	73	73
5	0	0	0	175	73	73
0	0	0	0	175	73	73
0	0	0	0	175	73	73
0	0	0	0	175	73	73
0	0	0	0	175	73	80
0	0		0	180	85	85
	0			180	85	73
				180	85	73
5	0	0	0	180	85	73
10	0	0	0	180	85	73
				180	85	78
0	0	0	0	180	85	89
0	0	0	0	180	85	89
0	0	0	0	180	85	89
				196	90	90
		0		196	90	
				196	90	
				196	90	
0	0		0			
	0					78
	0					78
	0					
	0					
0	0,03		0	178	68	68
5	0,03	0,06	0,03	178	68	78
0	0		0	183	86	86
5	0	0	0	183	86	78
	0			183	86	78
5	0	0	0	183	86	78
				183	86	86
5	0	0	0	183	86	86
5	0	0	0	183	86	86
	0			183	86	86
	0			183	86	
0	0	0	0	183	86	
20	0	0	0	183	86	
	0			183	86	
					59	59
					59	86
		0			59	
					59	
					59	
					59	
					59	
0	0		0	170	85	85
	0			170	85	86
	0			170	85	86
0	0		0		65	65
	0				65	86
0	0		0	186	80	80
	0			186	80	86
0	0	0	0	186	80	86
5	0	0	0	186	80	86
5	0	0	0	186	80	86
10	0	0	0	186	80	82
10	0	0	0	186	80	82
				187	84	84
				187	84	86
		0		187	84	86
				187	84	87
		0		187	84	87
		0		187	84	87
				187	84	87
				187	84	87
		0		187	84	87
				187	84	87
		0		187	84	
		0		187	84	
				187	84	
		0		187	84	
		0		187	84	
				187	84	
0	0,1		0	170	83	83
20	0,1	0,2	0,1	170	83	87
				170	83	93
-13	0,1			170	83	93
0	0,1	0,2	0,1	170	83	93
-30	0,1	0	-0,1	170	83	93
-30	0,1	0	-0,1	170	83	93
				170	83	91
-30	0,1	0	-0,1	170	83	91
-30	0,1	0	-0,1	170	83	95
-30	0,1	0	-0,1	170	83	95
0	0		0	175	61	61
	0			175	61	91
-5	0	0	0	175	61	91
				175	61	76
				175	61	64
0	0	0	0	175	61	64
				175	61	64
0	0	0	0	175	61	64
				175	61	65
-5	0	0	0	175	61	65
				175	61	61
	0			175	61	62
-5	0	0	0	175	61	62
-5	0	0	0	175	61	62
	0			175	61	62
-5	0	0	0	175	61	62
0	0,06		0	178	69	69
0	0,06	0	-0,06	178	69	61
	0,06			178	69	61
	0,06			178	69	61
	0,06			178	69	61
0	0		0	176	81	81
-5	0	0	0	176	81	61
-5	0	0	0	176	81	61
-5	0	0	0	176	81	61
-5	0	0	0	176	81	61
-5	0	0	0	176	81	61
				176	81	84
	0			176	81	84
-5	0	0	0	176	81	84
0	0		0	153	65	65
	0			153	65	84
0	0	0,03	0,03	153	65	84
0	0	0	0	153	65	84
10	0	0	0	153	65	84
-5	0	0	0	153	65	65
-10	0	0	0	153	65	61
-10	0	0,03	0,03	153	65	61
0	0		0	180	80	80
5	0	0	0	180	80	84
				180	80	74
	0			180	80	74
				180	80	75
-5	0	0	0	180	80	75
				180	80	76
	0			180	80	76
5	0	0	0	180	80	
				161	49	49
		0		161	49	76
		0		161	49	76
				161	49	49
		0		161	49	49
		0		161	49	49
		0		161	49	49
		0		161	49	49
		0		161	49	52
0	0		0	170	71	71
0	0	0	0	170	71	49
				170	71	72
5	0	0	0	170	71	72
0	0	0	0	170	71	
-5	0	0	0	170	71	
5	0	0	0	170	71	
0	0		0	162	64	64
-10	0	0	0	162	64	72
-5	0	0	0	162	64	72
				162	64	60
-10	0	0	0	162	64	60
-10	0	0	0	162	64	60
-5	0	0	0	162	64	60
	0			162	64	60
-10	0	0	0	162	64	60
-10	0	0	0	162	64	64
-10	0	0	0	162	64	64
-10	0	0	0	162	64	64
	0			162	64	64
				160	47	47
				160	47	60
		0		160	47	60
				160	47	46
		0		160	47	46
				160	47	46
		0		160	47	46
		0		160	47	46
				160	47	44
		0		160	47	44
				160	47	45
		0		160	47	45
				160	47	44
		0		160	47	44
		0		160	47	44
		0		160	47	45
0	0		0	180	74	74
	0			180	74	44
0	0	0,03	0,03	180	74	44
				180	74	72
5	0	0,03	0,03	180	74	72
	0			180	74	72
0	0	0,06	0,06	180	74	72
5	0	0,03	0,03	180	74	72
0	0	0,06	0,06	180	74	72
0	0	0,03	0,03	180	74	76
0	0	0	0	180	74	76
0	0	0,03	0,03	180	74	78
0	0		0	177	87	87
0	0	0	0	177	87	72
	0			177	87	85
5	0	0,03	0,03	177	87	85
	0			177	87	85
	0			177	87	85
	0			177	87	85
5	0	0,03	0,03	177	87	85
0	0	0	0	177	87	85
0	0	0	0	177	87	85
5	0	0	0	177	87	85
				177	87	95
	0			177	87	
	0			177	87	
0	0		0	170	66	66
-10	0	0	0	170	66	95
0	0,03		0	181	97	97
5	0,03	0,03	0	181	97	95
5	0,03	0	-0,03	181	97	95
				181	97	97
0	0,03	0	-0,03	181	97	97
	0,03			181	97	97
				181	97	98
5	0,03	0,03	0	181	97	98
0	0,03	0,03	0	181	97	98
0	0		0	168	53	53
	0			168	53	98
	0			168	53	98
0	0		0	160	87	87
0	0	0	0	160	87	98
				160	87	88
-10	0	0	0	160	87	88
-10	0	0	0	160	87	88
-10	0	0	0	160	87	88
				160	87	87
-10	0	0	0	160	87	87
-10	0	0	0	160	87	87
-10	0	0	0	160	87	87
				170		
				170		87
				170		57
				170		57
				170		55
				175		
				175		62
		0		175		62
		0		175		62
				175		66
		0		175		66
				175		67
				175		
		0,03		175		
0	0		0	176	78	78
-5	0	0	0	176	78	67
	0			176	78	78
				170		
				170		70
		0		170		70
				170		68
		0		170		68
		0		170		68
		0		170		71
