# Supplementary material for: Long-term efficacy and safety of rilpivirine plus abacavir and lamivudine in HIV-1 infected patients with undetectable viral load
Source: PLoS One. 2018 Feb 16;13(2):e0191300. doi: 10.1371/journal.pone.0191300 (PMC5815573; doi:10.1371/journal.pone.0191300)
Supplement: S4 Dataset — (PDF) [file pone.0191300.s004.pdf]

ID	SMOKE BASELINE	BLOOD PRESSURE, MAX	BLOOD PRESSURE, MIN	DIABETES
864	2	127	72	0
864	2	127	72	0
864	2	127	72	0
864	2	127	72	0
1161	1	140	70	1
1161	1	140	70	1
1161	1	140	70	1
1161	1	140	70	1
1161	1	140	70	1
1161	1	140	70	1
1161	1	140	70	1
1161	1	140	70	1
1161	1	140	70	1
1161	1	140	70	1
1161	1	140	70	1
1204	2	140	66	0
1204	2	140	66	0
1204	2	140	66	0
1204	2	140	66	0
1234	9	120	80	0
1234	9	120	80	0
1234	9	120	80	0
1234	9	120	80	0
1234	9	120	80	0
1250	2	110	70	0
1250	2	110	70	0
1250	2	110	70	0
1250	2	110	70	0
1250	2	110	70	0
1250	2	110	70	0
1250		110	70	0
1304	2	130	80	0
1304	2	130	80	0
1304	2	130	80	0
1304	2	130	80	0
1304	2	130	80	0
1304	2	130	80	0
1340	1	157	95	0
1340	1	157	95	0
1340	1	157	95	0
1340	1	157	95	0
1340	1	157	95	0
1340	1	157	95	0
1340	1	157	95	0
1622	2	115	84	0
1622	2	115	84	0
1622	2	115	84	0

1622	2	115	84	0
1622	2	115	84	0
1675	1	129	65	0
1675	1	129	65	0
1675	1	129	65	0
1675	1	129	65	0
1675	1	129	65	0
1675	1	129	65	0
1675	1	129	65	0
2075	9	120	80	0
2075	9	120	80	0
2075	9	120	80	0
2161	1	116	68	0
2161	1	116	68	0
2161	1	116	68	0
2161	1	116	68	0
2161	1	116	68	0
2161	1	116	68	0
2161	1	116	68	0
2194	2	140	90	0
2194	2	140	90	0
2194	2	140	90	0
2194	2	140	90	0
2194	2	140	90	0
2393	3	139	86	0
2393	3	139	86	0
2393	3	139	86	0
2393	3	139	86	0
2393	3	139	86	0
2484	2	120	78	0
2484	2	120	78	0
2484	2	120	78	0
2484	2	120	78	0
2484	2	120	78	0
2484	2	120	78	0
2641	3	130	90	1
2641	3	130	90	1
2641	3	130	90	1
2641	3	130	90	1
2641	3	130	90	1
2641	3	130	90	1
2641	3	130	90	1
2641	3	130	90	1
2641	3	130	90	1
2641	3	130	90	1
2641	3	130	90	1
2641	3	130	90	1
2641	3	130	90	1
2641	3	130	90	1
2641	3	130	90	1

2641	3	130	90	1
2641	3	130	90	1
2641	3	130	90	1
2890	2	135	88	0
2890	2	135	88	0
2890	2	135	88	0
2890	2	135	88	0
2890	2	135	88	0
2912	1	99	68	0
2912	1	99	68	0
2912	1	99	68	0
2912	1	99	68	0
2912	1	99	68	0
2912	1	99	68	0
2912	1	99	68	0
2912	1	99	68	0
2933	2	120	70	0
2933	2	120	70	0
3203	1	110	70	0
3203	1	110	70	0
3203	1	110	70	0
3203	1	110	70	0
3203	1	110	70	0
3203	1	110	70	0
3203	1	110	70	0
3203	1	110	70	0
3203	1	110	70	0
3203	1	110	70	0
3536	1	130	80	0
3536	1	130	80	0
3536	1	130	80	0
3536	1	130	80	0
3536	1	130	80	0
3574	1	120	80	0
3574	1	120	80	0
3713	9			0
3713	9			0
3713	9			0
3741	2	100	60	0
3741	2	100	60	0
3741	2	100	60	0
3741	2	100	60	0
3824	3	130	76	1
3824	3	130	76	1
3824	3	130	76	1
3824	3	130	76	1
3824	3	130	76	1
3824	3	130	76	1
3897	2	130	90	0
3897	2	130	90	0

3897	2	130	90	0
3897	2	130	90	0
3897	2	130	90	0
3897	2	130	90	0
3965	1	110	63	0
3965	1	110	63	0
4016	9			0
4016	9			0
4016	9			0
4018	2	134	86	0
4018	2	134	86	0
4018	2	134	86	0
4018	2	134	86	0
4018	2	134	86	0
4018	2	134	86	0
4018	2	134	86	0
4018	2	134	86	0
4054	2	113	65	0
4054	2	113	65	0
4054	2	113	65	0
4054	2	113	65	0
4054	2	113	65	0
4172	2	120	85	0
4172	2	120	85	0
4172	2	120	85	0
4172	2	120	85	0
4172	2	120	85	0
4172	2	120	85	0
4172	2	120	85	0
4172	2	120	85	0
4172	2	120	85	0
4172	2	120	85	0
4267	2	160	90	0
4267	2	160	90	0
4267	2	160	90	0
4267	2	160	90	0
4267	2	160	90	0
4267	2	160	90	0
4267	2	160	90	0
4267	2	160	90	0
4267	2	160	90	0
4267	2	160	90	0
4267	2	160	90	0
4267	2	160	90	0
4267	2	160	90	0
4267	2	160	90	0
4267	2	160	90	0
4279	1	123	78	0
4279	1	123	78	0
4279	1	123	78	0

4279	1	123	78	0
4279	1	123	78	0
4279	1	123	78	0
4279	1	123	78	0
4279	1	123	78	0
4282	2	91	64	0
4282	2	91	64	0
4282	2	91	64	0
4282	2	91	64	0
4282	2	91	64	0
4282	2	91	64	0
4282	2	91	64	0
4282	2	91	64	0
4282	2	91	64	0
4282	2	91	64	0
4668	1	136	84	0
4668	1	136	84	0
4668	1	136	84	0
4669	2	119	74	0
4669	2	119	74	0
4669	2	119	74	0
4669	2	119	74	0
4669	2	119	74	0
4669	2	119	74	0
4669	2	119	74	0
4669	2	119	74	0
4974	1	137	100	1
4974	1	137	100	1
4974	1	137	100	1
4974	1	137	100	1
4974	1	137	100	1
4974	1	137	100	1
4974	1	137	100	1
5070	1	110	70	0
5070	1	110	70	0
5070	1	110	70	0
5070	1	110	70	0
5133	2			0
5133	2			0
5133	2			0
5133	2			0
5133	2			0
5184	1	130	87	0
5184	1	130	87	0
5184	1	130	87	0
5184	1	130	87	0
5184	1	130	87	0
5184	1	130	87	0
5184	1	130	87	0
5184	1	130	87	0

5184	1	130	87	0
5184	1	130	87	0
5184	1	130	87	0
5184	1	130	87	0
5192	3	118	76	0
5192	3	118	76	0
5192	3	118	76	0
5192	3	118	76	0
5192	3	118	76	0
5192	3	118	76	0
5192	3	118	76	0
5192	3	118	76	0
5192	3	118	76	0
5192	3	118	76	0
5192	3	118	76	0
5192	3	118	76	0
5247	2	110	70	0
5247	2	110	70	0
5247	2	110	70	0
5247	2	110	70	0
5288	1	120	75	0
5288	1	120	75	0
5288	1	120	75	0
5336	2	133	81	0
5336	2	133	81	0
5336	2	133	81	0
5336	2	133	81	0
5336	2	133	81	0
5336	2	133	81	0
5336	2	133	81	0
5378	2	120	75	0
5378	2	120	75	0
5378	2	120	75	0
5378	2	120	75	0
5378	2	120	75	0
5378	2	120	75	0
5378	2	120	75	0
5378	2	120	75	0
5495	1	130	80	0
5495	1	130	80	0
5495	1	130	80	0
5495	1	130	80	0
5495	1	130	80	0
5495	1	130	80	0
5571	2	123	77	0
5571	2	123	77	0
5571	2	123	77	0
5571	2	123	77	0
5571	2	123	77	0
5571	2	123	77	0

5571	2	123	77	0
5571	2	123	77	0
5571	2	123	77	0
5571	2	123	77	0
5571	2	123	77	0
5571	2	123	77	0
5571	2	123	77	0
5571	2	123	77	0
5571	2	123	77	0
5571	2	123	77	0
5571	2	123	77	0
5687	3			0
5782	2	155	97	0
5782	2	155	97	0
5782	2	155	97	0
5782	2	155	97	0
5782	2	155	97	0
5868	2	130	82	0
5868	2	130	82	0
5868	2	130	82	0
5868	2	130	82	0
5868	2	130	82	0
6009	2	139	85	0
6009	2	139	85	0
6009	2	139	85	0
6009	2	139	85	0
6009	2	139	85	0
6009	2	139	85	0
6009	2	139	85	0
6034	2	125	75	0
6034	2	125	75	0
6034	2	125	75	0
6060	9	122	88	0
6060	9	122	88	0
6081	9	120	80	0
6081	9	120	80	0
6081	9	120	80	0
6081	9	120	80	0
6222	3	117	73	0
6222	3	117	73	0
6222	3	117	73	0
6222	3	117	73	0
6222	3	117	73	0
6307	1			0
6307	1			0
6433	2	120	80	0
6433	2	120	80	0
6433	2	120	80	0
6433	2	120	80	0
6433	2	120	80	0

6433	2	120	80	0
6433	2	120	80	0
6433	2	120	80	0
6461	2	122	90	0
6461	2	122	90	0
6461	2	122	90	0
6461	2	122	90	0
6461	2	122	90	0
6461	2	122	90	0
6461	2	122	90	0
6461	2	122	90	0
6461	2	122	90	0
6461	2	122	90	0
6461	2	122	90	0
6672	2	110	65	0
6672	2	110	65	0
6672	2	110	65	0
6672	2	110	65	0
6672	2	110	65	0
6672	2	110	65	0
6672	2	110	65	0
6672	2	110	65	0
6677	2	130	90	0
6677	2	130	90	0
6677	2	130	90	0
6677	2	130	90	0
6677	2	130	90	0
6680	2	134	75	0
6680	2	134	75	0
6680	2	134	75	0
6680	2	134	75	0
6680	2	134	75	0
6680	2	134	75	0
6680	2	134	75	0
6680	2	134	75	0
6680	2	134	75	0
6680	2	134	75	0
6680	2	134	75	0
6680	2	134	75	0
6680	2	134	75	0
6680	2	134	75	0
6680	2	134	75	0
6680	2	134	75	0
6680	2	134	75	0
6680	2	134	75	0
6751	1	154	111	0
6751	1	154	111	0
6751	1	154	111	0
6751	1	154	111	0
6769	9	117	78	0

6769	9	117	78	0
6769	9	117	78	0
6769	9	117	78	0
6769	9	117	78	0
6769	9	117	78	0
6790	3	137	89	0
6790	3	137	89	0
6790	3	137	89	0
6790	3	137	89	0
6790	3	137	89	0
6790	3	137	89	0
6790	3	137	89	0
6790	3	137	89	0
6790	3	137	89	0
6790	3	137	89	0
6790	3	137	89	0
6881	2	112	76	1
6881	2	112	76	1
6881	2	112	76	1
6944	2	116	79	0
6944	2	116	79	0
6944	2	116	79	0
6944	2	116	79	0
6944	2	116	79	0
6944	2	116	79	0
6944	2	116	79	0
6944	2	116	79	0
6944	2	116	79	0
6944	2	116	79	0
6947	2	120	80	0
6947	2	120	80	0
6947	2	120	80	0
6947	2	120	80	0
6947	2	120	80	0
6947	2	120	80	0
6953	1	110	70	0
6953	1	110	70	0
6953	1	110	70	0
6953	1	110	70	0
6955	1	105	71	0
6955	1	105	71	0
6955	1	105	71	0
6955	1	105	71	0
6955	1	105	71	0
6955	1	105	71	0
6955	1	105	71	0
6955	1	105	71	0
6955	1	105	71	0
6955	1	105	71	0
6955	1	105	71	0

6972	9	116	68	0
6972	9	116	68	0
6972	9	116	68	0
6972	9	116	68	0
7137	1	130	90	0
7137	1	130	90	0
7137	1	130	90	0
7137	1	130	90	0
7137	1	130	90	0
7137	1	130	90	0
7137	1	130	90	0
7137	1	130	90	0
7137	1	130	90	0
7137	1	130	90	0
7137	1	130	90	0
7145	1			0
7145	1			0
7145	1			0
7145	1			0
7145	1			0
7200	2	107	68	0
7200	2	107	68	0
7200	2	107	68	0
7200	2	107	68	0
7200	2	107	68	0
7245	2	119	73	0
7245	2	119	73	0
7245	2	119	73	0
7245	2	119	73	0
7245	2	119	73	0
7245	2	119	73	0
7245	2	119	73	0
7245	2	119	73	0
7245	2	119	73	0
7245	2	119	73	0
7245	2	119	73	0
7245	2	119	73	0
7245	2	119	73	0
7283	1	105	63	0
7283	1	105	63	0
7408	2	121	74	0
7408	2	121	74	0
7756	1	113	70	0
7756	1	113	70	0
7756	1	113	70	0
7756	1	113	70	0
7756	1	113	70	0
7756	1	113	70	0
7756	1	113	70	0
7756	1	113	70	0

7756	1	113	70	0
7756	1	113	70	0
7756	1	113	70	0
7756	1	113	70	0
7853	2	120	75	0
7853	2	120	75	0
7853	2	120	75	0
7853	2	120	75	0
7853	2	120	75	0
7853	2	120	75	0
7853	2	120	75	0
7853	2	120	75	0
7853	2	120	75	0
7853	2	120	75	0
7918	1	120	80	0
7918	1	120	80	0
7918	1	120	80	0
7918	1	120	80	0
7935	2	110	90	0
7935	2	110	90	0
7935	2	110	90	0
7935	2	110	90	0
8063	1	120	75	0
8063	1	120	75	0
8063	1	120	75	0
8063	1	120	75	0
8063	1	120	75	0
8063	1	120	75	0
8063	1	120	75	0
8063	1	120	75	0
8063	1	120	75	0
8103	1	120	70	1
8103	1	120	70	1
8103	1	120	70	1
8103	1	120	70	1
8103	1	120	70	1
8103	1	120	70	1
8103	1	120	70	1
8118	1	130	90	0
8118	1	130	90	0
8118	1	130	90	0
8118	1	130	90	0
8118	1	130	90	0
8153	1	130	80	0
8153	1	130	80	0
8153	1	130	80	0
8153	1	130	80	0
8153	1	130	80	0
8248	2	120	70	0
8248	2	120	70	0

8248	2	120	70	0
8248	2	120	70	0
8248	2	120	70	0
8248	2	120	70	0
8328	3	112	66	0
8328	3	112	66	0
8328	3	112	66	0
8328	3	112	66	0
8328	3	112	66	0
8328	3	112	66	0
8328	3	112	66	0
8334	2	125	82	0
8481	9			0
8481	9			0
8481	9			0
8490	1	144	87	0
8490	1	144	87	0
8575	1	112	72	0
8575	1	112	72	0
8575	1	112	72	0
8575	1	112	72	0
8575	1	112	72	0
8575	1	112	72	0
8575	1	112	72	0
8575	1	112	72	0
8575	1	112	72	0
8575	1	112	72	0
8575	1	112	72	0
8797	2	124	77	0
8797	2	124	77	0
8824	9	138	80	0
8824	9	138	80	0
8824	9	138	80	0
8833	1	120	85	0
8833	1	120	85	0
8855	2	129	95	0
8855	2	129	95	0
8855	2	129	95	0
8855	2	129	95	0
8855	2	129	95	0
8855	2	129	95	0
8855	2	129	95	0
8863	1	130	75	0
8863	1	130	75	0
8863	1	130	75	0
8863	1	130	75	0
8863	1	130	75	0
8863	1	130	75	0
8863	1	130	75	0
8863	1	130	75	0

8863	1	130	75	0
8863	1	130	75	0
8863	1	130	75	0
8863	1	130	75	0
8863	1	130	75	0
8873	2	120	75	0
8873	2	120	75	0
8873	2	120	75	0
8873	2	120	75	0
8873	2	120	75	0
8873	2	120	75	0
8873	2	120	75	0
8873	2	120	75	0
8873	2	120	75	0
8873	2	120	75	0
8965	2	120	80	0
8965	2	120	80	0
8965	2	120	80	0
8965	2	120	80	0
8965	2	120	80	0
8965	2	120	80	0
8965	2	120	80	0
8965	2	120	80	0
8965	2	120	80	0
8965	2	120	80	0
8965	2	120	80	0
9005	1	125	80	0
9005	1	125	80	0
9005	1	125	80	0
9005	1	125	80	0
9005	1	125	80	0
9081	3	126	90	0
9081	3	126	90	0
9081	3	126	90	0
9081	3	126	90	0
9081	3	126	90	0
9081	3	126	90	0
9081	3	126	90	0
9081	3	126	90	0
9081	3	126	90	0
9156	2	126	84	0
9156	2	126	84	0
9156	2	126	84	0
9156	2	126	84	0
9156	2	126	84	0
9156	2	126	84	0
9156	2	126	84	0
9156	2	126	84	0
9395	2	122	77	0
9395	2	122	77	0

9395	2	122	77	0
9395	2	122	77	0
9395	2	122	77	0
9395	2	122	77	0
9395	2	122	77	0
9395	2	122	77	0
9395	2	122	77	0
9424	1	110	70	0
9424	1	110	70	0
9424	1	110	70	0
9424	1	110	70	0
9424	1	110	70	0
9424	1	110	70	0
9424	1	110	70	0
9424	1	110	70	0
9424	1	110	70	0
9452	9	160	115	0
9452	9	160	115	0
9452	9	160	115	0
9452	9	160	115	0
9452	9	160	115	0
9452	9	160	115	0
9452	9	160	115	0
9456	1	130	80	0
9456	1	130	80	0
9456	1	130	80	0
9456	1	130	80	0
9456	1	130	80	0
9456	1	130	80	0
9456	1	130	80	0
9456	1	130	80	0
9456	1	130	80	0
9456	1	130	80	0
9456	1	130	80	0
9456	1	130	80	0
9479	1	120	70	0
9479	1	120	70	0
9479	1	120	70	0
9479	1	120	70	0
9479	1	120	70	0
9479	1	120	70	0
9479	1	120	70	0
9479	1	120	70	0
9479	1	120	70	0
9479	1	120	70	0
9479	1	120	70	0
9479	1	120	70	0
9479	1	120	70	0
9479	1	120	70	0
9479	1	120	70	0

9483	1	140	90	1
9483	1	140	90	1
9483	1	140	90	1
9483	1	140	90	1
9483	1	140	90	1
9483	1	140	90	1
9483	1	140	90	1
9483	1	140	90	1
9483	1	140	90	1
9483	1	140	90	1
9483	1	140	90	1
9483	1	140	90	1
9502	2	130	90	0
9502	2	130	90	0
9502	2	130	90	0
9502	2	130	90	0
9502	2	130	90	0
9502	2	130	90	0
9502	2	130	90	0
9502	2	130	90	0
9502	2	130	90	0
9502	2	130	90	0
9502	2	130	90	0
9502	2	130	90	0
9561	2	145	88	0
9561	2	145	88	0
9587	2	120	80	0
9587	2	120	80	0
9587	2	120	80	0
9587	2	120	80	0
9587	2	120	80	0
9587	2	120	80	0
9587	2	120	80	0
9587	2	120	80	0
9587	2	120	80	0
9637	1	111	78	0
9637	1	111	78	0
9637	1	111	78	0
9682	2	150	82	0
9682	2	150	82	0
9682	2	150	82	0
9682	2	150	82	0
9682	2	150	82	0
9682	2	150	82	0
9682	2	150	82	0
9682	2	150	82	0
9682	2	150	82	0
9901	2	109	74	0
9901	2	109	74	0
9901	2	109	74	0

9901	2	109	74	0
9901	2	109	74	0
9955	1	113	70	0
9955	1	113	70	0
9955	1	113	70	0
9955	1	113	70	0
9955	1	113	70	0
9955	1	113	70	0
9955	1	113	70	0
10057	9	130	80	0
10057	9	130	80	0
10057	9	130	80	0
10355	9			0
10355	9			0
10355	9			0
10355	9			0
10355				0

HOMA BASELINE	HOMA	INSULIN BASELINE	INSULIN
0,96		4,9	
0,96		4,9	
0,96		4,9	
0,96		4,9	

1,76		8,3	
1,76		8,3	
1,76		8,3	
1,76		8,3	
1,88		10,2	
1,88		10,2	
1,88		10,2	
1,88	1,13	10,2	6
1,88		10,2	
1,88		10,2	
1,88	2,43	10,2	11,9
1,88		10,2	
1,88	2,72	10,2	13,3
1,88	2,43	10,2	11,5
1,88		10,2	
1,88	2,57	10,2	12
3,52		15,2	
3,52		15,2	
3,52		15,2	
3,52	2,88	15,2	13,6
3,52		15,2	
3,52	2,35	15,2	10,4
0,95		4,7	
0,95		4,7	
0,95		4,7	
0,95		4,7	
0,95		4,7	
0,95		4,7	
0,95		4,7	
1,03		4,7	
1,03		4,7	
1,03		4,7	

1,03	2,68	4,7	13,8
1,03		4,7	
0,88		3,9	
0,88		3,9	
0,88	1,3	3,9	6,1
0,88		3,9	
0,88		3,9	
0,88	1,27	3,9	5,4
0,88	2,74	3,9	10,1
1,18		6,3	
1,18		6,3	
1,18		6,3	
0,9		4,2	
0,9		4,2	
0,9		4,2	
0,9		4,2	
0,9		4,2	
0,9		4,2	
0,9		4,2	
2,6		12	
2,6		12	
2,6		12	
2,6	5,05	12	23,3
2,6	2,99	12	14,1
1,25		6,2	
1,25		6,2	
1,25		6,2	
1,25		6,2	
1,25		6,2	

26,16		62,4	
26,16		62,4	
26,16		62,4	
26,16		62,4	
26,16		62,4	
26,16		62,4	
26,16		62,4	
26,16		62,4	
26,16		62,4	
26,16		62,4	
26,16		62,4	
26,16	9,54	62,4	22,5
26,16		62,4	
26,16		62,4	
26,16		62,4	

26,16		62,4	
26,16	12,33	62,4	23,6
26,16	11,54	62,4	32,5
1,32		5,3	
1,32		5,3	
1,32		5,3	
1,32		5,3	
1,32	1,18	5,3	6,1
0,95		4,1	
0,95		4,1	
0,95		4,1	
0,95		4,1	
0,95		4,1	
0,95		4,1	
0,95		4,1	
0,95		4,1	
2,51		12,9	
2,51		12,9	
1,95		9,2	
1,95		9,2	
1,95		9,2	
1,95		9,2	
1,95		9,2	
1,95	2,94	9,2	15,9
1,95		9,2	
1,95		9,2	
1,95	3,58	9,2	15
1,95	4,38	9,2	21,4
1,77		9,6	
1,77		9,6	
1,77		9,6	
1,77		9,6	
1,77		9,6	
0,47		2,5	
0,47		2,5	
1,23		5,8	
1,23		5,8	
1,23	2	5,8	9,7
1,59		7,6	
1,59		7,6	
1,59		7,6	
1,59		7,6	
2,89		8,1	
2,89		8,1	
2,89		8,1	
2,89		8,1	
2,89		8,1	
2,89		8,1	

1,15		5,4	
1,15		5,4	
1,36		6,2	
1,36		6,2	
1,36		6,2	
1,67		8,1	
1,67		8,1	
1,67		8,1	
1,67	2,03	8,1	9,7
1,67		8,1	
1,67		8,1	
1,67		8,1	
1,67		8,1	
1,19		5,5	
1,19		5,5	
1,19		5,5	
1,19		5,5	
1,19		5,5	
0,87		4,1	
0,87	0,95	4,1	4,9
0,87	1,11	4,1	5,8
0,87		4,1	
0,87	0,65	4,1	3,3
0,87		4,1	
0,87	0,69	4,1	3,6
0,87	0,85	4,1	3,9
0,87	1,5	4,1	7,1
0,87	1,13	4,1	5,5
1,13		4,7	
1,13		4,7	
1,13	1,98	4,7	7,3
1,13		4,7	
1,13		4,7	
1,13	2,2	4,7	7,5
1,13	2,01	4,7	6,3
1,13		4,7	
1,13		4,7	
1,13	2,62	4,7	8,5
1,13		4,7	
1,13	2,07	4,7	6,9
1,13	2,34	4,7	10
1,13	2,39	4,7	8,2
1,13	2,02	4,7	6,2
0,28		1,7	
0,28		1,7	
0,28		1,7	

0,28		1,7	
0,28		1,7	
0,28		1,7	
0,28		1,7	
0,28		1,7	
3,69		13,5	
3,69	1,13	13,5	4,9
3,69		13,5	
3,69	0,69	13,5	3,2
3,69		13,5	
3,69		13,5	
3,69		13,5	
3,69		13,5	
3,69	0,94	13,5	4,1
3,69		13,5	
5,04		33	
5,04		33	
5,04		33	
2,27		11	
2,27		11	
2,27		11	
2,27		11	
2,27		11	
2,27		11	
2,27		11	
2,27		11	
4,37		10,5	
4,37		10,5	
4,37		10,5	
4,37	8,64	10,5	15,1
4,37		10,5	
4,37	9,86	10,5	21,5
4,37	8,17	10,5	14,6
1,62		7,4	
1,62	2,17	7,4	8,9
1,62	1,23	7,4	6,5
1,62	1,7	7,4	8,1
0,81		3,4	
0,81		3,4	
0,81	0,51	3,4	2,7
0,81		3,4	
0,81		3,4	
1,46		7,9	
1,46		7,9	
1,46		7,9	
1,46	2,91	7,9	13,6
1,46		7,9	
1,46	1,81	7,9	10,2
1,46		7,9	
1,46	3,47	7,9	15,3

1,46	3,05	7,9	15,3
1,46	4,05	7,9	19,8
1,46	4,91	7,9	23,4
1,46	3,12	7,9	16
1,92		8,2	
1,92		8,2	
1,92		8,2	
1,92		8,2	
1,92		8,2	
1,92	1,51	8,2	7,6
1,92		8,2	
1,92		8,2	
1,92		8,2	
1,92		8,2	
1,92		8,2	
1,92		8,2	
1,01		5,3	
1,01		5,3	
1,01		5,3	
1,01		5,3	
1,75		8,7	
1,75		8,7	
1,75		8,7	
	0,69		3,6
1		4,5	
1		4,5	
1		4,5	
1		4,5	
1		4,5	
1		4,5	
1		4,5	
1		4,5	
0,99		4,3	
0,99	1,73	4,3	7,5
0,99	0,95	4,3	4,6
0,99		4,3	
0,99	1,68	4,3	7,1
0,99		4,3	
1,39		6,8	
1,39		6,8	
1,39		6,8	
1,39		6,8	
1,39		6,8	
1,39		6,8	

1,39		6,8	
1,39		6,8	
1,39		6,8	
1,39		6,8	
1,39		6,8	
1,39	2,71	6,8	13,6
1,39		6,8	
1,39	1,86	6,8	10,1
1,39		6,8	
1,39		6,8	
1,39	1,6	6,8	7,4
3,9		19,3	
1,27		5,4	
1,27		5,4	
1,27		5,4	
1,27		5,4	
1,27		5,4	
2,27		12	
2,27		12	
2,27		12	
2,27	4,66	12	21
2,27		12	
1,75		7,9	
1,75		7,9	
1,75		7,9	
1,75		7,9	
1,75		7,9	
1,75		7,9	
1,75		7,9	
2,75		9,9	
2,75		9,9	
2,75	1,65	9,9	6,2
2,07		10,9	
2,07		10,9	
1,77		7	
1,77		7	
1,77		7	
1,77		7	
0,98		5,1	
0,98		5,1	
0,98		5,1	
0,98		5,1	
0,98		5,1	
1,81		10,5	
1,81	0,66	10,5	3,5
0,55		2,6	
0,55		2,6	
0,55	0,46	2,6	2,1
0,55		2,6	
0,55	0,23	2,6	1,3

0,55		2,6	
0,55		2,6	
0,55	0,31	2,6	1,8
1,39		6,8	
1,39		6,8	
1,39	1,17	6,8	6,2
1,39	1,11	6,8	6,1
1,39		6,8	
1,39		6,8	
1,39		6,8	
1,39	1,22	6,8	6,3
1,39	0,84	6,8	4,8
1,39	1,32	6,8	6,1
1,39	1,58	6,8	8,2
1,59		9,4	
1,59		9,4	
1,59		9,4	
1,59		9,4	
1,59		9,4	
1,59		9,4	
1,59		9,4	
1,59	1,88	9,4	9
0,76		3,8	
0,76		3,8	
0,76	0,67	3,8	3,4
0,76		3,8	
0,76	0,76	3,8	4
4,7		24,8	
4,7	12,11	24,8	51,7
4,7		24,8	
4,7	8,87	24,8	40
4,7		24,8	
4,7		24,8	
4,7	1,24	24,8	6,3
4,7		24,8	
4,7		24,8	
4,7		24,8	
4,7		24,8	
4,7		24,8	
4,7	1,26	24,8	6,4
4,7		24,8	
4,7	1,49	24,8	7,4
4,7		24,8	
4,7	1,33	24,8	7,4
4,7	25,39	24,8	90,3
1,47		6,3	
1,47		6,3	
1,47		6,3	
1,47		6,3	
4,08		17,8	

4,08		17,8	
4,08		17,8	
4,08		17,8	
4,08		17,8	
4,08		17,8	
7,26		18,3	
7,26		18,3	
7,26	9,95	18,3	20,8
7,26		18,3	
7,26	4,26	18,3	14,3
7,26	5,81	18,3	16,5
7,26	5,19	18,3	12,4
7,26	6,48	18,3	17,4
7,26	6,52	18,3	17,4
7,26	6,53	18,3	19,2
7,26	6,23	18,3	15,5
3,85		12,5	
3,85		12,5	
3,85		12,5	
0,86		4,8	
0,86		4,8	
0,86		4,8	
0,86		4,8	
0,86	1,26	4,8	7
0,86		4,8	
0,86	1,51	4,8	7,7
0,86	1,33	4,8	6,2
0,86	0,96	4,8	5,1
0,86	1,54	4,8	8,2
0,7		3,9	
0,7	0,64	3,9	3,3
0,7	0,77	3,9	4
0,7	1,14	3,9	5,6
0,7	0,54	3,9	2,9
0,7	0,84	3,9	4,5
0,84		4,2	
0,84		4,2	
0,84		4,2	
0,84		4,2	
0,66		3,4	
0,66		3,4	
0,66		3,4	
0,66		3,4	
0,66		3,4	
0,66	0,69	3,4	3,8
0,66	0,67	3,4	3,4
0,66		3,4	
0,66	0,36	3,4	2,3
0,66	0,99	3,4	5,4
0,66	1,08	3,4	5,4

0,61		3,1	
0,61		3,1	
0,61		3,1	
0,61		3,1	
1,87		8	
1,87		8	
1,87		8	
1,87	2,37	8	11,6
1,87		8	
1,87	1,57	8	7,6
1,87		8	
1,87	2,25	8	10,5
1,87		8	
1,87	2,59	8	12,4
1,87		8	
0,73		3,7	
0,73	0,59	3,7	3,4
0,73		3,7	
0,73		3,7	
0,73		3,7	
0,95		4,4	
0,95		4,4	
0,95		4,4	
0,95		4,4	
0,95		4,4	
1,24		5,1	
1,24		5,1	
1,24		5,1	
1,24		5,1	
1,24		5,1	
1,24	0,41	5,1	2,1
1,24		5,1	
1,24	1,2	5,1	6,1
1,24	1,45	5,1	6,7
1,24	0,87	5,1	4,4
1,24	1,1	5,1	5,2
1,24	1,66	5,1	7,3
1,24	1,26	5,1	5,8
1,72		9,6	
1,72		9,6	
0,92		4,4	
0,92		4,4	
0,83		3,7	
0,83	1,73	3,7	8
0,83		3,7	
0,83		3,7	
0,83		3,7	
0,83		3,7	
0,83		3,7	
0,83		3,7	

0,83		3,7	
0,83		3,7	
0,83		3,7	
0,83		3,7	
22,32		37,4	
22,32		37,4	
22,32		37,4	
22,32		37,4	
22,32		37,4	
22,32		37,4	
22,32		37,4	
22,32		37,4	
22,32		37,4	
22,32		37,4	
1,55		7,1	
1,55	0,99	7,1	4,5
1,55	2,49	7,1	10,2
1,55	1,47	7,1	6,2
1,19		5,9	
1,19	0,57	5,9	3,3
1,19		5,9	
1,19		5,9	
2,26		10,3	
2,26		10,3	
2,26		10,3	
2,26		10,3	
2,26		10,3	
2,26		10,3	
2,26		10,3	
2,26		10,3	
2,26		10,3	
5,91		20	
5,91		20	
5,91		20	
5,91		20	
5,91		20	
5,91		20	
5,91		20	
2,14		9,9	
2,14	1,23	9,9	5,8
2,14	2,47	9,9	11,8
2,14		9,9	
2,14		9,9	
0,94		4,2	
0,94		4,2	
0,94		4,2	
0,94		4,2	
0,94		4,2	
4,43		9,1	
4,43		9,1	

4,43		9,1	
4,43	7,78	9,1	38,5
4,43	2,29	9,1	12,4
4,43	3,48	9,1	19,9
0,35		2	
0,35		2	
0,35		2	
0,35		2	
0,35		2	
0,35		2	
0,35	1,67	2	7,9
1,11		5,3	
2,07		9,8	
2,07		9,8	
2,07		9,8	
1,05		4,8	
1,05	1,8	4,8	8,7
1,51		7,8	
1,51	1,28	7,8	6
1,51	0,57	7,8	4,7
1,51	2,15	7,8	9,4
1,51		7,8	
1,51	1,82	7,8	8,3
1,51	0,95	7,8	4
1,51	1,73	7,8	8
1,51	0,56	7,8	3
1,51	1,14	7,8	6,9
1,51	1,79	7,8	9,2
1,1		5,1	
1,1		5,1	
0,91		4,6	
0,91		4,6	
0,91		4,6	
1,65		8,7	
1,65		8,7	
1,85		9,5	
1,85		9,5	
1,85	0,97	9,5	6,1
1,85	0,89	9,5	4,9
1,85	1,58	9,5	7,4
1,85	1,73	9,5	8,1
1,85	1,62	9,5	8,2
0,68		3,8	
0,68		3,8	
0,68		3,8	
0,68		3,8	2,3
0,68		3,8	
0,68	0,85	3,8	3,7
0,68		3,8	
0,68		3,8	

0,68	0,36	3,8	1,7
0,68		3,8	
0,68	1	3,8	4,6
0,68	1,01	3,8	4,8
0,68	1,1	3,8	5,2
2,21		12,3	
2,21		12,3	
2,21		12,3	
2,21		12,3	
2,21		12,3	
2,21		12,3	
2,21		12,3	
2,21		12,3	
2,21	3,18	12,3	13,9
2,21	3,11	12,3	19,4
1,76		9,2	
1,76		9,2	
1,76		9,2	
1,76		9,2	
1,76		9,2	
1,76		9,2	
1,76		9,2	
1,76	1,91	9,2	10,5
1,76		9,2	
1,76	2,19	9,2	11
1,76		9,2	
1,13		5,1	
1,13		5,1	
1,13		5,1	
1,13		5,1	
1,13		5,1	
1,88		9,1	
1,88		9,1	
1,88	9,7	9,1	41,4
1,88	1,31	9,1	7,4
1,88	1,36	9,1	7,7
1,88	1,97	9,1	10
1,88		9,1	
1,88		9,1	
1,88	5,23	9,1	26,2
1,6		6,7	
1,6		6,7	
1,6	1,55	6,7	6,5
1,6	2,2	6,7	9,7
1,6	2,73	6,7	11,8
1,6	1,61	6,7	7,6
1,6	3,63	6,7	14,9
1,6	1,48	6,7	6,6
1,27		6	
1,27	0,96	6	5,1

1,27		6	
1,27		6	
1,27		6	
1,27		6	
1,27		6	
1,27		6	
1,27	1,32	6	6,7
0,97		4,5	
0,97	1,17	4,5	4,6
0,97	0,81	4,5	3,4
0,97		4,5	
0,97	0,95	4,5	4,1
0,97	0,58	4,5	2,6
0,97	0,63	4,5	2,7
0,97	1,37	4,5	4,9
0,97	0,94	4,5	3,7
1,73		7,5	
1,73	3,07	7,5	14
1,73		7,5	
1,73	2,03	7,5	9,5
1,73	3,8	7,5	16,6
1,73	2,64	7,5	11,3
1,73	6,6	7,5	27,3
2,36		12,3	
2,36	1,84	12,3	9
2,36	2,01	12,3	10,2
2,36		12,3	
2,36	2,29	12,3	11,5
2,36	3,33	12,3	16,1
2,36	2,99	12,3	14,8
2,36		12,3	
2,36	1,01	12,3	7,2
2,36	1,93	12,3	8,8
2,36	1,89	12,3	9,8
2,36	1,93	12,3	9,9
3,07		15,8	
3,07		15,8	
3,07		15,8	
3,07		15,8	
3,07		15,8	
3,07		15,8	
3,07	0,97	15,8	4,9
3,07	1	15,8	5,3
3,07		15,8	
3,07	1,49	15,8	6,8
3,07		15,8	
3,07	0,53	15,8	2,8
3,07		15,8	
3,07	1,4	15,8	6,1
3,07	0,78	15,8	3,7

2,34		12,5	
2,34		12,5	
2,34	2,67	12,5	14,1
2,34		12,5	
2,34	3,01	12,5	15,1
2,34		12,5	
2,34	2,42	12,5	12
2,34	3,15	12,5	16,2
2,34	3,17	12,5	15,9
2,34	3,74	12,5	18,1
2,34	3,09	12,5	17,2
2,34	4,87	12,5	21
1,36		6,9	
1,36		6,9	
1,36		6,9	
1,36		6,9	
1,36		6,9	
1,36		6,9	
1,36		6,9	
1,36		6,9	
1,36		6,9	
1,36		6,9	
1,36		6,9	
1,36		6,9	
1,08		4,6	
1,08		4,6	
5,29		14,8	
5,29		14,8	
5,29	3,58	14,8	10,7
5,29		14,8	
5,29	1,61	14,8	4,4
5,29		14,8	
5,29		14,8	
5,29		14,8	
5,29	1,51	14,8	5
0,54		3,8	
0,54		3,8	
0,54	8,75	3,8	40,8
3,25		13,6	
3,25	1,93	13,6	8,1
3,25		13,6	
3,25	2,77	13,6	10,5
3,25		13,6	
3,25	3,93	13,6	16,8
3,25		13,6	
3,25	4,99	13,6	17,9
3,25	2,99	13,6	11,9
	1,18		6

	2,96		13,2
	0,94		4,8
	1,01		5,3
	1,26		6,2
1,63		7,9	
1,63		7,9	
1,63		7,9	
	1,63		8
	2,09		10,6
